# Local and long-distance migration among young people in rural Malawi: importance of age, sex and family

**DOI:** 10.12688/wellcomeopenres.19309.1

**Published:** 2023-05-11

**Authors:** Estelle McLean, Albert Dube, Fredrick Kalobekamo, Emma Slaymaker, Amelia C Crampin, Rebecca Sear

**Affiliations:** 1 Epidemiology and Population Health, London School of Hygiene and Tropical Medicine, London, UK; 2 Malawi Epidemiology and Intervention Research Unit, Lilongwe, Malawi

**Keywords:** Children, adolescents, migration, mobility, Malawi, multi-level modelling, family

## Abstract

*Background*

In sub-Saharan Africa, migration of young people is common and occurs for a variety of reasons. Research focus is often on international or long-distance internal migration; however, shorter moves also affect people’s lives and can reveal important information about cultures and societies. In rural sub-Saharan Africa, migration may be influenced by cultural norms and family considerations: these may be changing due to demographic shifts, urbanisation, and increased media access.

*Methods*

We used longitudinal data from a Health and Demographic Surveillance Site in rural northern Malawi to present a detailed description of short and long distance, independent and accompanied, migration in young people between 2004-2017. We further explore the family and household factors which are associated with these moves using multi-level multinomial logistic regression modelling.

*Results & conclusions*

We found two key periods of mobility: in very young childhood, and in adolescence/young adulthood. In this traditionally patrilocal area, we found that young women move longer distances to live with their spouse. Despite the local patrilineal customs, we found evidence of the importance of the maternal family, and that female and male children may be treated differently from as young as age four, with girls more likely to migrate long distances independently, and more likely to accompany their mothers in other moves. Young people living close to relatives tend to have lower chances of moving, and those from more advantaged families are more likely to move.

## Introduction

Research in sub-Saharan Africa has found relatively high rates of mobility/migration in young children and adolescents/young adults ( Beegle & Poulin, 2013; Ford & Hosegood, 2005; Grieger *et al.*, 2013). Children and adolescents may move with their parents/guardians, and adolescents/young adults can be expected to move away from their natal home to live independently. Dependent children or adolescents may also move without their parents or guardians: children in sub-Saharan Africa may be fostered out to other households either temporarily or long-term, so that the fostering household may provide care for them or give them better educational opportunities, or so they can provide support to the fostering household through house/farm work ( Hedges *et al.*, 2019) or caring responsibilities ( Robson, 2000). Fostering may happen in a planned way, or in more emergency circumstances due to parental illness or death. Older children/adolescents may be sent to live in other households where there might be better able to work and earn wages to send back home ( Kwankye, 2012; Temin *et al.*, 2013).

On a population level, it is important to study migration to understand population flows and predict needs for services. It is also important to understand the effects of migration on individuals and communities as both positive and negative outcomes have previously been found: Migration can give people opportunities for education, work and to form new relationships: for adolescents in some communities, a period of migration has become a common part of transition to adulthood, where they can learn new skills and enjoy their independence ( Hertrich & Lesclingand, 2013); However, migration, particularly in unaccompanied children and adolescents, is often regarded in a negative light, as migrants may be more likely to drop out of school ( Clark & Cotton, 2013; Hashim, 2007), experience increased work load ( Hashim, 2007), social isolation ( Temin *et al.*, 2013), pre-marital pregnancy ( Xu *et al.*, 2013) and HIV ( Anglewicz & Reniers, 2014) (though it has been suggested that vulnerabilities associated with migration also lead to increased risk of HIV ( Magadi, 2013)); in Uganda, for both young men and women, migration was associated with greater chance of recent alcohol use and increased likelihood of engaging in risky sexual behaviour ( Schuyler *et al.*, 2017).

Migration is often studied in respect to international movements, and if internal movements are considered then often only long-distance moves are counted. Moving locally compared to long distance may have fewer consequences for people and the local environment: a qualitative study in Malawi found that when asked about moving house, children did not tend to report on short moves when they stayed with family or moved to a different family member, and the authors speculated that this could be because these moves did not change their lives much ( Young *et al.*, 2006). However, a local move is still a potentially disruptive event which may have both positive and negative effects ( van Blerk & Ansell, 2006) on a young person’s life, and short-distance migrations can also give valuable insights into local cultures and customs. In Malawi, international migration, in particular to South Africa, was common in colonial times but was discouraged from the 1960s ( Beegle & Poulin, 2013). While in recent years international migration has increased once more, internal migration is far more common ( Beegle & Poulin, 2013), and a high level of circular migration has been found among adolescents ( Chalasani *et al.*, 2013).

### Theoretical background and literature review

Theories of migration have been developed over the past several decades which attempt to explain migration patterns through economic, demographic, human capital and risk diversification lenses (see Piguet, 2018 for a summary). Many general concepts can be applied to the migration of children and adolescents in sub-Saharan Africa, in particular the New Economics of Labour Migration theory, which places the household rather than the individual as the decision-making unit ( Stark & Bloom, 1985). There are a few specific points to note regarding this household-based framework when conceptualising migration of young people in rural sub-Saharan Africa. Firstly, there is the level of agency that children have over household decisions that affect them. Of course, decision-making power may not be equally spread among household members of any age, but this is particularly the case among children, whose agency within the household depends on multiple factors including age and sex (boys may have more say in migration decisions than girls ( Hunleth *et al.*, 2015)). While the adults in the household are likely to make the final decision over the migration of a child or young adolescent, it would be incorrect to assume that they have no agency in the decision-making process: for example, after a divorce children may decide themselves who to accompany or may attempt to negotiate staying with relatives in the neighbourhood even if their parents move. Equally those children ‘sent’ away may be given different levels of say over this decision, even if the initial idea was not theirs ( Hashim, 2007). The second factor is related to adolescents’ desire for independence, and how the timing or format of this may be at odds with the desires of the other household members. Whitehead and colleagues suggest that migration in children and adolescents, particularly independent migration, can be viewed through the lens of the intergenerational contract: shared understandings between family members of what may be expected from each other ( Whitehead *et al.*, 2005). Thirdly, defining a household is not straightforward and is subject to cultural influences ( Randall *et al.*, 2011); this has been shown to affect understanding of migration in Nepal ( Agergaard, 1999). Finally, extended family is important in many cultures and it is likely that migration decisions will be made taking into account not just the immediate household or family, but other family members. Research in Zambia suggest that, rather than migration being the result of the household deciding what is best for all members, it can often be the result of conflicts over land and resources, leading to rupture in the household ( Cliggett, 2000).

Research on mobility and migration in young people in Africa tends to find differences by sex. Female children have been found to be more likely to be fostered out in research in Tanzania ( Hedges *et al.*, 2019) and Zimbabwe ( Robson, 2000). In Zambia, a study looking at short-term movements, found that girls were more likely to spend the holidays with extended kin, and also more likely than boys to have the reason for going as ‘helping with household chores’; boys were more likely to report ‘getting to know relatives’ though this was by far the most common response for both sexes ( Hunleth *et al.*, 2015). In general, adolescent girls and young women tend to be more likely to move: in Kenya, young migrants to urban areas were more likely to be female ( Clark & Cotton, 2013), in Malawi a study of young people aged 15–24 found that 47% of women had ever moved compared to 38% of men ( Beegle & Poulin, 2013). There are also differences found in the reason given for the move: young women tend to report moving for marriage, and men for work, education or economic reasons ( Anglewicz & Reniers, 2014; Beegle & Poulin, 2013; Chalasani *et al.*, 2013; Clark & Cotton, 2013) however it has been suggested that sex differences in reporting reasons for move may simply reflect the gender norms of the society, *i.e.,* it may not be socially acceptable for a woman to report moving for economic reasons, so she reports marriage even if her main consideration was economic, and vice versa for men ( Temin *et al.*, 2013). Despite these reporting biases, economic migration in young women has been seen to be just as common as in men in South Africa ( Camlin *et al.*, 2014) and in many West African countries a period of migration has been part of the transition to adulthood with young women usually spending time in the city before returning for marriage. This phenomena tends to be viewed differently for boys than girls, with girls facing more reluctance from their elders to let them go, the authors speculate that migration in young males is in line with family expectations ( *i.e.* to provide) so is accepted, while for women it is perceived that it mostly benefits the individual rather than the family ( Lesclingand & Hertrich, 2017), despite many young women report using the time to learn domestic skills and earn money to buy items to help them in their marital home ( Hertrich & Lesclingand, 2013).

Socioeconomic position has also been found to be associated with moving, though it seems to have a complex relationship with migration, with evidence that the most and least disadvantaged groups are most likely to move ( Ginsburg *et al.*, 2009). In Malawi, those in wealthier households were more likely to move unless they were farming families ( Beegle & Poulin, 2013). In Senegal, young people whose fathers had more education or higher socio-economic position in childhood were more likely move to urban areas, but less likely to move to rural ones: however this was influenced by the initial location, with the author reporting that access to more and better community resources makes later moves less likely ( Herrera-Almanza & Sahn, 2020). In Malawi, young movers were more likely to come from households with more assets ( Chalasani *et al.*, 2013).

Several studies have also examined parental presence, vital and marital status and household composition and found these to be associated with youth migration: children were found to be more likely to move if not living with their mother ( Madhavan *et al.*, 2012) and adolescents were more likely to move if the household head was not a parent ( Beegle & Poulin, 2013; Chalasani *et al.*, 2013). Loss of parent has also been found to increase migration for young women in Senegal ( Herrera-Almanza & Sahn, 2020), but in Malawi this effect was only seen in men ( Beegle & Poulin, 2013). While most studies have focused on parents, some have attempted to look beyond this *i.e.* Clark & Cotton in Kenya asked young respondents to indicate who was responsible for them, rather than making assumptions (2013) and a Malawian study asked generally about family and friends in the area. This latter study found that knowing family and friends prior to moving was associated with a longer length of stay at that location, but especially for women, knowing friends there was the most strongly associated ( Myroniuk, 2018). In many sub-Saharan African societies, the extended family is important in day-to-day life and is likely to impact decision making around migration.

### Aims of this paper

This analysis aims to provide a detailed description of mobility and migration in children and adolescents/young adults in rural Malawi, and to assess the role of family (within and outside the household) on accompanied and independent, long-distance and local migration in the same population using multinomial multi-level regression modelling. The data is from the Karonga Health and Demographic Surveillance Site from 2004–2017 and allows examination of the presence of different types of family members both in the household and living nearby: we have previously shown that young people in this population tend to live near extended family ( McLean *et al.*, 2021a).

## Methods

### Context

The Karonga Health and Demographic Surveillance Site (HDSS) was established in 2002 in the southern part of the Karonga district in northern Malawi ( Crampin *et al.*, 2012) by the Malawi Epidemiology and Intervention Research Unit (MEIRU- formerly known as the Karonga Prevention Study). The area is largely rural with one semi-urban trading town, several smaller market villages and one port on Lake Malawi. The majority of the population engage in subsistence farming or fishing. The main ethnic group are Tumbuka, who have followed patrilineal and patrilocal custom since the 19th century: women tend to move to their husband’s village when they marry ( Malawi Human Rights Commission, 2006). In the event of divorce or even paternal death, children considered to be old enough to be away from their mother may be required to live with their father’s family ( Malawi Human Rights Commission, 2006). Polygyny is widespread: at the end of 2016 about 15% of households in the HDSS were headed by men with more than one wife.

The HDSS covers an area of 150km ^2^ and by 2016 had over 40,000 people under surveillance, with very high response rates. Household membership is defined by the participants with guidance from trained fieldworkers: all household members must usually live in the dwelling/compound together and recognise the same household head. Men with more than one wife who do not live in the same location are assigned to be living in each wife’s household; all other individuals may only belong to one household. Households are identified by two unique numbers: one which does not change through the lifetime of the household (known as unique household ID for this analysis) and one which is related to the household’s location, which may change over time (known as geographic household ID for this analysis); GPS coordinates are recorded for each household when they are registered and if they move.

Births and deaths are captured monthly through a system of local ‘key informants’, while migrations are captured annually through visits to all households: information is gathered on any new or departed household member including date of move, reason for move and where they moved from/to. If a whole household moves, then this information is gathered from the key informants. When a new household member is registered, through birth or in-migration, where possible, members of any age are linked to their parents’ identification numbers if they have ever been assigned one (even if they are not currently HDSS participants). On an annual basis, participants are asked about their marital status and to provide information about their spouse(s): where possible, the identification numbers of the spouses have also been linked. This information was used to identify all family links (by blood and by marriage) between all HDSS participants.

### Ethics

Household heads provide written informed consent on behalf of the whole household to participate in the Karonga HDSS, which may be rescinded at any time for any reason. The HDSS is regularly reviewed and approved by the Malawian National Health Sciences Review Committee (approval #419), and the London School of Hygiene and Tropical Medicine Ethics Committee (approval #5081).

### Reproducibility

The data manipulation and analysis described below was carried out using Stata v16.1, and R v4.2.1 and MLwiN v3.05 (the latter two where indicated). R and MLwiN are freely available while a licence is required for Stata, however all techniques carried out in Stata for this paper may be reproduced in similar software, such as R.

### Dataset

Data on HDSS participants are gathered as event reports and surveys. The event data is used to create continuous episodes, and the survey data are assumed to be valid for dates within certain periods before and/or after the survey date (the length of these periods depend on the type of data). Generation some of the exposure variables involves calculating the distance between each index and multiple relatives and would be computationally intensive to apply to the longitudinal dataset so the episodic data were reduced to one data point per quarter (15th of the middle month of each quarter) per person, and the data treated as panel data. Each data point included variables indicating whether a person was living with, or within 250 metres of, specific types of family member, various indicators of socio-economic status, and information about the local area.

Moves were identified as individuals who had a different geographic household ID to the following quarterly snapshot, or if they were not present in the HDSS in the next quarter and were recorded to have migrated out, or if they were not present the previous quarter and were recorded as migrating in. Moves of less than five metres were dropped as these were likely to be artefacts of a new geographic household ID being assigned if a new household head was declared. If there was more than one move associated with a quarter ( *i.e.* a move in from outside of the area, and then a move out) then one move was kept randomly. Due to some disruptions to data collection in recent years, including due to Covid-19, only data up to the end of 2017 was used for this analysis.

### Exploratory analyses

The analysis can be divided into three main steps: exploratory, descriptive and regression analyses. *A priori*, it was decided to analyse the moves (1) by distance, (2) whether they were independent or accompanied, and by (3) age and (4) sex. The cut-offs/definitions used were defined during the exploratory analysis as described below.

### Distance

When a person moved within the HDSS area the actual distance moved was calculated using household coordinates, which are available for all. When the move was to or from an area external to the HDSS, in almost all cases the source or destination was gathered: for a town or city in Malawi, GPS coordinates from a central area of the town/city were used, for outside of Malawi, GPS coordinates of a point in the new country nearest to Malawi were used. This means that our analysis includes both internal and international migration, though the majority of moves are internal. To define the cut-off between short and long moves, a preliminary analysis of primary school attended before and after a move was carried out. Data are captured annually to record school grade and school name for school attenders: records of all primary school attenders were examined if they were still attending primary school and had a different geographic household ID ( *i.e.* had moved house) at the following interview. The distance between the households of the first and second interview was calculated and the averages compared between those who changed school and those who remained at the same school. There were 3916 record pairs from 3010 individuals which met these criteria. 2490 did not involve a change in school and the mean distance moved was 0.9km (95% CI allowing for clustering by individual ID 0.86-1.03), 1426 did change school and the mean distance was 4.6 (95% CI allowing for clustering by individual ID 4.4-4.8). Based on these results, it was decided to categorise short move as less than four kilometres and long moves as four kilometres or more.

### Independence

To identify whether the person moved alone or with members of their household, all household members were assessed to see whether they stayed in the original house, moved with the index person, or moved elsewhere. For external moves they were classed as moving together if they reported the same source or destination town or country, for moves within the HDSS they had to have the same destination household ID. People were then classed as moving independently (without a parent of any age, or an adult aged 18 or over) or accompanied (with at least one adult aged 18 or over, or a parent who may be under 18).

### Age/sex

The above definitions were applied to the panel dataset so that each record had an outcome of either ‘no move’ or one of four move types (short independent, long independent, short accompanied or long accompanied). The risk of each move outcome was calculated for each age year, separated by sex. Although the focus of the analysis was on young people, a high upper age limit of 34 was used initially, to be sure of observing the whole of the adolescent/young adult time. 95% confidence limits were calculated allowing for clustering within unique household ID and unique individual ID. Following these initial analyses (which are described in the results section), age was categorised as ‘children’ for females if under the age of 12 and for males if under 16 and ‘adolescents’ for females if aged 12–24 and males 16–28. The age ranges are different as females tend to experience transitions to adulthood earlier than males in this area ( McLean *et al.*, 2021b); even though the age ranges extend beyond typical definitions of adolescence, the term adolescent will be used for simplicity throughout.

***Descriptive comparison of family/household composition of movers by sex*.** For this analysis, the panel dataset was modified to only include records with a move outcome. Whether the household composition (the make-up of the household members in terms of their relationship to the index mover) differed between the sending and receiving household was examined using only short moves, as these are most likely to have full information on both sending and receiving household (as most long moves were to or from outside of the area). Firstly, the numbers moving between different household types were displayed on Sankey diagrams (created using the networkD3 package in R ( Gandrud *et al.*, 2017)) separately for children and adolescents, male and female, and independent and accompanied moves. The proportions in each category of the sending and receiving households were then compared by sex, separately by move type and age group, using Wald tests allowing for clustering within unique household ID and unique individual ID. The household composition variable was created using latent class analysis which has been described elsewhere ( McLean *et al.*, 2021a) with the following categories:

- Parents & siblings – both parents present, plus siblings aged under 18 if any, and does not fit into any of the below categories
- Sister's family – at least one sister aged over 18 years or her family, (more of sister+family present than brother+family if also present), and mother and/or father present or no maternal or paternal family present
- Brother's family – as above but with brother instead of sister
- Mother & siblings – mother present, no father present, nor father's other wife nor maternal family
- Father & stepmother – mother not present, father or father's other wife present
- Maternal – father and father's other wife not present, at least one maternal relative present and more maternal relatives present than paternal
- Paternal – mother and father's other wife not present, at least one paternal relative present and more paternal relatives present than maternal
- Spouse – spouse present
- Other – does not fit into any of the above categories
- External – household composition unknown as outside of HDSS area
- No IDs – household composition unknown as parent IDs unknown

In addition, whether children and adolescents were moving with their parents was examined using all short and long accompanied moves: the proportions in each category (no parents, mother only, father only, both) were compared by sex, separately by move type and age group using Wald tests allowing for clustering within unique household ID and unique individual ID.

***Regression analysis of associations between mobility and family and household composition/structure*.** For this analysis, the full panel dataset was used, including those who didn’t move. However, moves with no information on the ‘sending household’, *i.e.,* those migrating from outside the HDSS, were dropped, and, as the focus was on the effect of family, participants who had no record of either parent ID were excluded. Multi-level multinomial logit regression models allowing for clustering by unique household ID and unique individual ID were run on the full panel dataset using MLwiN Version 3.05 ( Charlton *et al.*, 2020), with the outcome of move type (no move [baseline], short independent, long independent, short accompanied and long accompanied) and including the family variables and potential confounders. The model was run separately for each age group and sex. The potential confounders were chosen due to existing literature having demonstrated associations with mobility (see introduction), and data availability ( *i.e.* further household socio-economic status variables could not be included due to lack of complete data). The variables included in this analysis were:

- Time-varying variables relating to family and household composition/structure:
  - A detailed household composition variable (as described above).
  - Number of people in the household in different age groups (under one year, one-four years, five-11 years, 12–18 years, 19–29 years, 30–59 years and 60 years and over) (all continuous variables which exclude the index person)
  - A total of four binary variables indicating presence of at least one relative within 250 metres but not in the immediate household from five family types: maternal (not including mother), paternal (not including father), sister’s (including sister aged 18 or over, but not younger sister), brother’s (including brother aged 18 or over, but not younger brother) and nuclear (parents and siblings aged under 18). The distance of 250 metres was chosen as it was assumed that relatives living closely are likely to be seeing each other regularly and have some influence on each other’s lives.

- Potential confounding variables:
  - Presence of own child in the household: to allow assessment of presence of small children after accounting for own child as having an own child may affect migration decisions (only for the adolescent analyses)
  - Whether biological mother is known to be dead (vital status of parents is derived directly from the HDSS record of the parent, or from information gathered on each participant’s parents at the annual survey)
  - Whether biological father is known to be dead
  - Age
  - Year (in two-year bands)
  - Distance to tarmac road,
  - Population density within 250 metres (categorised),
  - Whether father had any secondary education,
  - Whether mother had any secondary education
  - Employment ranking of the household head (categorised into low, which include not working or precariously employed, medium which includes subsistence farming and fishing, and high which includes waged employment and business owning)

## Results

**Figure 1.**
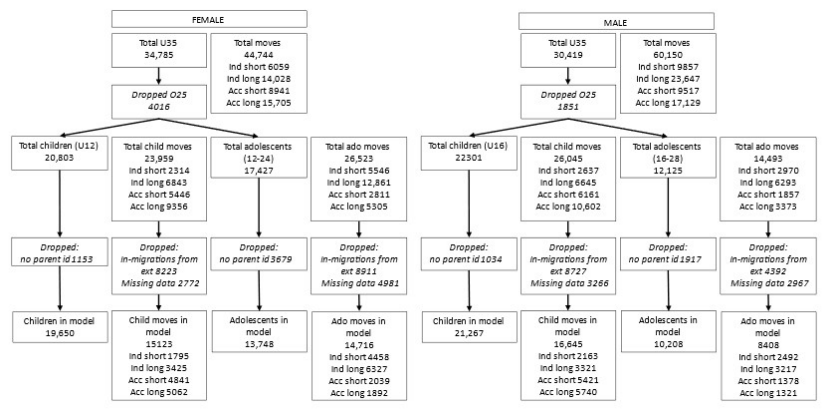
Number of participants and moves at different stages of analysis.

A total of 65,204 (34,785 female and 30,419 male) individuals aged under 35 contributed data to the initial exploratory analysis. There were 104,883 (44,733 female and 60,150 male) moves: 15,916 short independent; 37,675 long independent; 18,458 short accompanied and 32,834 long accompanied ( Figure 1).

### Percentage who moves by age and sex

**Figure 2.**
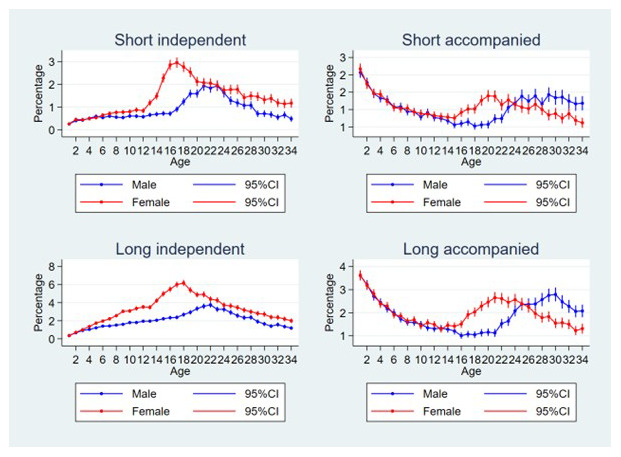
Percentage of individuals experiencing each move type at difference ages, by sex. Short moves are under 4 kilometres; independent moves are without a parent of any age or an adult aged 18 or over; male data is blue, female is red; note that each figure has a different scale.

Plots of the percentage of individuals that experienced each move type (short independent, long independent, short accompanied and long accompanied) at difference ages, by sex can be seen in Figure 2. For short independent moves, the risk increased sharply for females from the age of 12 up to age 17 before falling less steeply; for males the risks only increased from age 16, peaking at age 21–23, however risks were almost always lower than for females. For long independent moves for both sexes, the risks gradually increased during childhood, for females the risk was higher than for males from the age of four onwards, and the increase became steeper age 12 with a peak at age 18; for males the peak was at age 22. For short, accompanied moves, the risks were highest in the youngest children (risks for females and males are similar at very young age) which then declined to age 14–15, from here the risks increased again for females to a peak at age 20, while for males the risks increased again to age 24 and then remained stable or declined. The pattern was similar for long accompanied moves, except that for males the risks continued to increase up to age 30, before declining.

### Comparison of family/household composition of movers by sex

A total of 43,104 (20,803 females aged under 12, and 22,301 males aged under 16) children and 29,552 (17,427 females aged 12–24 and 12,125 males aged 16–28) adolescents contributed data to the descriptive analyses. There were 50,004 (23,959 female and 26,045 male) child moves (4951 short independent; 13,488 long independent; 11,607 short accompanied and 19,958 long accompanied) and 41,016 (26,523 female and 14,493 male) adolescent moves (8516 short independent; 19,154 long independent; 4668 short accompanied and 8678 long accompanied) ( Figure 1).

## Changing household composition

**Figure 3.**
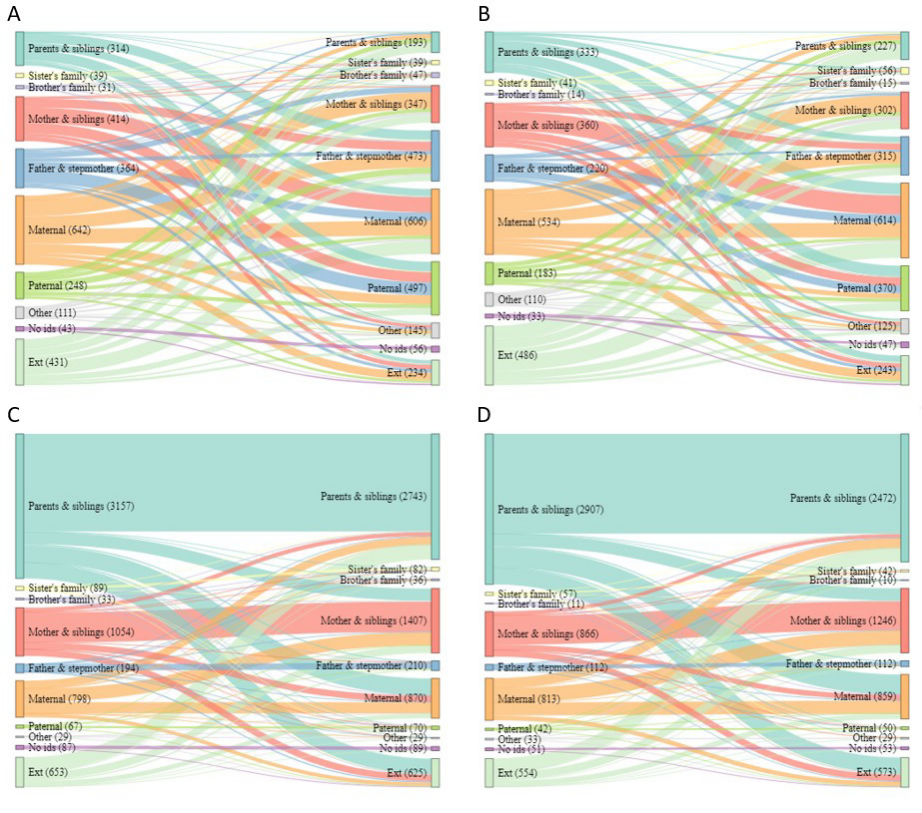
Sankey diagrams showing flow between sending and receiving households for all short moves by children. Short moves are under 4 kilometres; independent moves are without a parent of any age or an adult aged 18 or over; part **A** shows independent moves by male children (aged <16), **B** independent moves by female children (aged <12); **C** accompanied moves by male children; **D** accompanied moves by female children.

**Figure 4.**
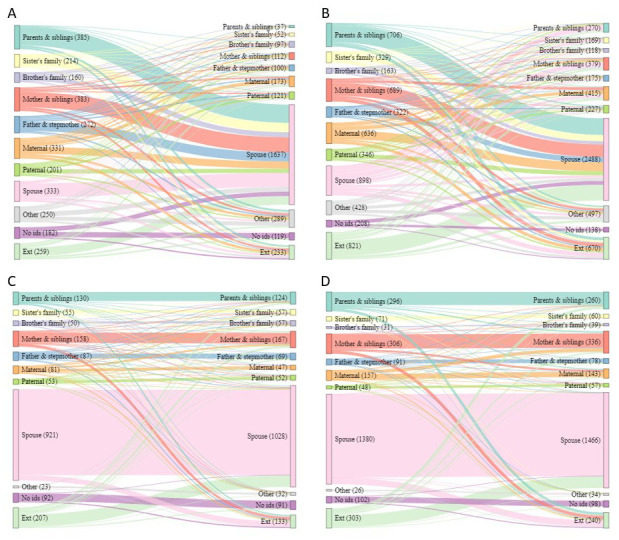
Sankey diagrams showing flow between sending and receiving households for all short moves by adolescents. Short moves are under 4 kilometres; independent moves are without a parent of any age or an adult aged 18 or over; part **A** shows independent moves by male adolescents (aged 16–28), **B** independent moves by female adolescents (aged 12–24); **C** accompanied moves by male adolescents; **D** accompanied moves by female adolescents.

Sankey diagrams showing the household composition of sending and receiving households shown in Figure 3 and Figure 4 show the difference in types of short move for children and adolescents (respectively). For children moving independently, there was not one more common move type, and children mostly changed household type when they moved ( Figure 3A & B). By sex, there was some evidence that female children were more likely to move to or from ‘ *parents and siblings’*, to ‘ *sister’s family*’ or ‘ *maternal’* or from outside of the area, while male children were more likely to move to or from ‘ *brother’s family’*, ‘ *father & stepmother’* and ‘ *paternal’* ( Table 1). For accompanied children, moving from a ‘ *parents and siblings’* household to another of the same type was most common, moving to and from ‘ *mother and siblings’* and ‘ *maternal’* households was also common ( Figure 3C & D). By sex, there was some evidence that male children were more likely to move to or from ‘ *sister’s family’*, ‘ *brother’s family’* and ‘ *father and stepmother’*, while female children were more likely to move to or from ‘ *maternal’* ( Table 1). For adolescents, the picture is different, for both male and female adolescents moving independently there was a wide variety of sending households, however the destination household were overwhelmingly ‘ *spouse’* households. A visible difference between males and females in this group is that females moving from ‘ *spouse’* households go to a wider variety of receiving households (‘ *parents and siblings’*, ‘ *mother and siblings’* etc.) ( Figure 4A & B). By sex, female adolescents were more likely to move to ‘ *parents and siblings’*, ‘ *sister’s family’*, ‘ *mother and siblings*’, ‘ *maternal’* and from or to outside of the area. Female adolescents were more likely to move from ‘ *spouse’* households, but male adolescents were more likely to move to ‘ *spouse’* households ( Table 1). The accompanied moves for adolescents show a different picture again, with the majority moving from a ‘ *spouse’* household to another of the same type ( Figure 4C & D). By sex, female adolescents were more likely to move from and to ‘ *parents and siblings’*, ‘ *mother and siblings*’ and ‘ *maternal’* households, while male adolescents were more likely to move from and to ‘ *brother’s family*’ ( Table 1).

**Table 1.** Sending or receiving household type by move type, age and sex, short moves only. Short moves are under 4 kilometres; independent moves are without a parent of any age or an adult aged 18 or over; female children are aged <12, male children are aged <16, female adolescents are aged 12-24 and male adolescents are aged 16-28; confidence intervals and p-values calculated allowing for clustering by unique household id and unique individual id.

	Sending household									Receiving household								
	Female				Male				p	Female				Male				p
	n	%	95% CI		n	%	95% CI			n	%	95% CI		n	%	95% CI		
*Child independent*																		
Parents & siblings	333	14.4%	12.7%	16.1%	314	11.9%	10.4%	13.5%	0.011	227	9.8%	8.5%	11.1%	193	7.3%	6.1%	8.5%	0.003
Sister's family	41	1.8%	1.2%	2.3%	39	1.5%	1.0%	2.0%	0.432	56	2.4%	1.8%	3.1%	39	1.5%	1.0%	2.0%	0.026
Brother's family	14	0.6%	0.3%	0.9%	31	1.2%	0.7%	1.6%	0.014	15	0.6%	0.3%	1.0%	47	1.8%	1.2%	2.3%	<0.001
Mother & siblings	360	15.6%	13.8%	17.3%	414	15.7%	14.0%	17.4%	0.895	302	13.1%	11.5%	14.6%	347	13.2%	11.7%	14.6%	0.912
Father & stepmother	220	9.5%	8.1%	10.9%	364	13.8%	12.2%	15.4%	<0.001	315	13.6%	12.0%	15.2%	473	17.9%	16.2%	19.7%	<0.001
Maternal	534	23.1%	21.1%	25.1%	642	24.3%	22.4%	26.3%	0.310	614	26.5%	24.5%	28.6%	606	23.0%	21.2%	24.8%	0.004
Paternal	183	7.9%	6.7%	9.2%	248	9.4%	8.1%	10.7%	0.067	370	16.0%	14.3%	17.7%	497	18.8%	17.1%	20.6%	0.011
Other	110	4.8%	3.8%	5.7%	111	4.2%	3.4%	5.0%	0.367	125	5.4%	4.4%	6.4%	145	5.5%	4.5%	6.5%	0.889
No ids	33	1.4%	0.9%	2.0%	43	1.6%	1.1%	2.1%	0.586	47	2.0%	1.4%	2.7%	56	2.1%	1.5%	2.7%	0.832
Ext	486	21.0%	19.3%	22.7%	431	16.3%	14.8%	17.9%	<0.001	243	10.5%	9.2%	11.8%	234	8.9%	7.7%	10.0%	0.045
*Child accompanied*																		
Parents & siblings	2907	53.4%	51.5%	55.3%	3157	51.2%	49.4%	53.1%	0.044	2472	45.4%	43.5%	47.3%	2743	44.5%	42.6%	46.4%	0.407
Sister's family	57	1.0%	0.7%	1.4%	89	1.4%	1.0%	1.9%	0.056	42	0.8%	0.5%	1.1%	82	1.3%	0.9%	1.8%	0.005
Brother's family	11	0.2%	0.1%	0.3%	33	0.5%	0.3%	0.8%	0.009	10	0.2%	0.1%	0.3%	36	0.6%	0.3%	0.8%	0.001
Mother & siblings	866	15.9%	14.5%	17.3%	1054	17.1%	15.8%	18.5%	0.133	1246	22.9%	21.3%	24.4%	1407	22.8%	21.3%	24.4%	0.962
Father & stepmother	112	2.1%	1.6%	2.5%	194	3.1%	2.6%	3.7%	0.001	112	2.1%	1.6%	2.5%	210	3.4%	2.8%	4.0%	<0.001
Maternal	813	14.9%	13.6%	16.3%	798	13.0%	11.8%	14.1%	0.005	859	15.8%	14.5%	17.0%	870	14.1%	13.0%	15.3%	0.020
Paternal	42	0.8%	0.5%	1.0%	67	1.1%	0.7%	1.5%	0.167	50	0.9%	0.6%	1.2%	70	1.1%	0.8%	1.5%	0.334
Other	33	0.6%	0.3%	0.9%	29	0.5%	0.3%	0.7%	0.422	29	0.5%	0.3%	0.8%	29	0.5%	0.3%	0.6%	0.685
No ids	51	0.9%	0.7%	1.2%	87	1.4%	1.0%	1.8%	0.044	53	1.0%	0.7%	1.3%	89	1.4%	1.0%	1.8%	0.057
Ext	554	10.2%	9.2%	11.2%	653	10.6%	9.6%	11.6%	0.443	573	10.5%	9.5%	11.6%	625	10.1%	9.1%	11.2%	0.504
*Adolescent independent*																		
Parents & siblings	706	12.7%	11.7%	13.8%	385	13.0%	11.6%	14.3%	0.767	270	4.9%	4.3%	5.4%	37	1.2%	0.8%	1.7%	<0.001
Sister's family	329	5.9%	5.2%	6.7%	214	7.2%	6.1%	8.3%	0.036	169	3.0%	2.6%	3.5%	52	1.8%	1.3%	2.2%	<0.001
Brother's family	163	2.9%	2.4%	3.4%	160	5.4%	4.5%	6.3%	<0.001	118	2.1%	1.7%	2.5%	97	3.3%	2.6%	3.9%	0.003
Mother & siblings	689	12.4%	11.4%	13.5%	383	12.9%	11.5%	14.3%	0.561	379	6.8%	6.1%	7.5%	112	3.8%	3.1%	4.5%	<0.001
Father & stepmother	322	5.8%	5.1%	6.5%	272	9.2%	8.0%	10.3%	<0.001	175	3.2%	2.7%	3.6%	100	3.4%	2.7%	4.0%	0.599
Maternal	636	11.5%	10.5%	12.4%	331	11.1%	9.9%	12.4%	0.668	415	7.5%	6.8%	8.2%	173	5.8%	4.9%	6.7%	0.003
Paternal	346	6.2%	5.5%	7.0%	201	6.8%	5.7%	7.8%	0.381	227	4.1%	3.6%	4.6%	121	4.1%	3.3%	4.8%	0.968
Spouse	898	16.2%	15.2%	17.2%	333	11.2%	10.0%	12.4%	<0.001	2488	44.9%	43.5%	46.2%	1637	55.1%	53.2%	57.0%	<0.001
Other	428	7.7%	7.0%	8.5%	250	8.4%	7.4%	9.5%	0.280	497	9.0%	8.2%	9.7%	289	9.7%	8.6%	10.8%	0.267
No ids	208	3.8%	3.2%	4.3%	182	6.1%	5.2%	7.0%	<0.001	138	2.5%	2.1%	2.9%	119	4.0%	3.2%	4.8%	0.001
Ext	821	14.8%	13.9%	15.7%	259	8.7%	7.7%	9.7%	<0.001	670	12.1%	11.2%	13.0%	233	7.8%	6.9%	8.8%	<0.001
*Adolescent accompanied*																		
Parents & siblings	296	10.5%	9.1%	12.0%	130	7.0%	5.5%	8.5%	<0.001	260	9.2%	7.9%	10.6%	124	6.7%	5.2%	8.1%	0.002
Sister's family	71	2.5%	1.8%	3.2%	55	3.0%	2.0%	3.9%	0.418	60	2.1%	1.4%	2.8%	57	3.1%	2.1%	4.1%	0.107
Brother's family	31	1.1%	0.6%	1.6%	50	2.7%	1.8%	3.6%	0.002	39	1.4%	0.9%	1.9%	57	3.1%	2.2%	4.0%	0.001
Mother & siblings	306	10.9%	9.5%	12.3%	158	8.5%	6.9%	10.1%	0.011	336	12.0%	10.5%	13.4%	167	9.0%	7.5%	10.5%	0.001
Father & stepmother	91	3.2%	2.4%	4.1%	87	4.7%	3.4%	6.0%	0.040	78	2.8%	2.0%	3.5%	69	3.7%	2.6%	4.8%	0.115
Maternal	157	5.6%	4.6%	6.6%	81	4.4%	3.3%	5.5%	0.082	143	5.1%	4.1%	6.0%	47	2.5%	1.7%	3.3%	<0.001
Paternal	48	1.7%	1.2%	2.3%	53	2.9%	2.0%	3.8%	0.027	57	2.0%	1.4%	2.6%	52	2.8%	2.0%	3.6%	0.129
Spouse	1380	49.1%	46.8%	51.4%	921	49.6%	46.9%	52.3%	0.703	1466	52.2%	49.9%	54.4%	1028	55.4%	52.6%	58.1%	0.020
Other	26	0.9%	0.5%	1.4%	23	1.2%	0.6%	1.9%	0.444	34	1.2%	0.7%	1.7%	32	1.7%	0.9%	2.5%	0.267
No ids	102	3.6%	2.9%	4.4%	92	5.0%	3.8%	6.2%	0.054	98	3.5%	2.7%	4.3%	91	4.9%	3.7%	6.1%	0.044
Ext	303	10.8%	9.5%	12.0%	207	11.1%	9.6%	12.7%	0.651	240	8.5%	7.4%	9.7%	133	7.2%	5.8%	8.5%	0.049

## Moving with parents

The proportion of accompanied movers according to whether they moved with their parents is shown in Table 2. Males tended to be more likely than females to move with neither parent; females were more likely to move with just mother, and male children with just their father (though this was not common); female adolescents were more likely to move with both parents for short moves.

**Table 2.** Whether moved with parents by move type (accompanied only), length and sex. Short moves are under 4 kilometres; independent moves are without a parent of any age or an adult aged 18 or over; female children are aged <12, male children are aged <16, female adolescents are aged 12–28 and male adolescents are aged 16–24; confidence intervals and p-values calculated allowing for clustering by unique household id and unique individual id.

	Short moves									Long moves								
	Female				Male				p	Female				Male				p
	n	%	95% CI		n	%	95% CI			n	%	95% CI		n	%	95% CI		
*Child accompanied*																		
Neither	323	5.9%	5.2%	6.7%	454	7.4%	6.5%	8.2%	0.005	816	8.7%	8.0%	9.4%	997	9.4%	8.7%	10.1%	0.132
Mother only	2652	48.7%	46.7%	50.6%	2831	46.0%	44.0%	47.9%	0.011	4145	44.3%	42.8%	45.8%	4368	41.2%	39.8%	42.6%	<0.001
Father only	121	2.2%	1.7%	2.7%	223	3.6%	3.0%	4.3%	<0.001	262	2.8%	2.4%	3.2%	401	3.8%	3.3%	4.3%	0.001
Both parents	2350	43.2%	41.2%	45.1%	2653	43.1%	41.1%	45.0%	0.934	4133	44.2%	42.7%	45.7%	4836	45.6%	44.1%	47.1%	0.088
*Adolescent accompanied*																		
Neither	1947	69.3%	67.1%	71.4%	1425	76.7%	74.3%	79.2%	<0.001	3914	73.8%	72.1%	75.4%	2636	78.1%	76.3%	80.0%	<0.001
Mother only	469	16.7%	15.0%	18.4%	217	11.7%	9.9%	13.5%	<0.001	534	10.1%	9.0%	11.1%	222	6.6%	5.5%	7.7%	<0.001
Father only	80	2.8%	2.1%	3.6%	62	3.3%	2.3%	4.4%	0.407	141	2.7%	2.1%	3.2%	111	3.3%	2.5%	4.1%	0.149
Both parents	315	11.2%	9.6%	12.8%	153	8.2%	6.6%	9.9%	0.002	716	13.5%	12.1%	14.8%	404	12.0%	10.4%	13.5%	0.056

### Regression analysis of association of family and household composition/structure with mobility

A total of 7783 (1153 female children, 1034 male children, 3679 female adolescents and 1917 male adolescents) were dropped from the regression analysis as they had no parent identifiers, so family variables could not be constructed; 36,128 moves were dropped either as well they were moves from outside of the DSS so did not have information on the sending household/area or because the mover did not have parent IDs ( Figure 1). The full results from the models, including confounding variables, are displayed in the Table 3– Table 6, while figures displaying the key results from the three family and household composition/structure variables plus selected other factors (highlighted as they showed interesting associations) are discussed below.

**Table 3.** Results for short independent and long independent move outcome for children, from multi-level multinomial regression models. Models run separately for female children (aged <12 [19,650 individuals]) and male children (aged <16 [21,267 individuals]); outcome is move type with ‘no move’ as baseline; models allow for clustering by unique household id and unique participant id; short moves are under 4 kilometres; independent moves are without a parent of any age or an adult aged 18 or over.

	Children (short independent moves)												Children (long independent moves)											
	Female (1795 moves)						Male (2163 moves)						Female (3425 moves)						Male (3321 moves)					
	OR	95% CI		SE	Z	p	OR	95% CI		SE	Z	p	OR	95% CI		SE	Z	p	OR	95% CI		SE	Z	p
Household composition																								
*Parents & siblings*	Reference						Reference						Reference						Reference					
*Sister's family*	3.1	2.2	4.5	0.18	6.3	<0.001	2.9	2.0	4.1	0.18	5.9	<0.001	2.4	1.9	2.9	0.11	8.0	<0.001	2.1	1.7	2.7	0.13	5.9	<0.001
*Brother's family*	8.0	4.4	14.6	0.31	6.8	<0.001	9.2	6.0	14.1	0.22	10.2	<0.001	5.0	3.2	7.6	0.22	7.3	<0.001	6.7	4.9	9.2	0.16	11.8	<0.001
*Mother & siblings*	4.0	3.3	4.7	0.09	15.2	<0.001	4.3	3.6	5.1	0.09	16.7	<0.001	2.5	2.2	2.8	0.07	14.1	<0.001	3.9	3.4	4.4	0.07	19.6	<0.001
*Father & stepmother*	8.0	6.6	9.8	0.10	20.6	<0.001	9.4	7.9	11.1	0.09	25.3	<0.001	4.8	4.1	5.5	0.07	21.3	<0.001	6.3	5.4	7.3	0.07	25.0	<0.001
*Maternal*	6.4	5.3	7.8	0.10	19.3	<0.001	8.7	7.3	10.5	0.09	23.3	<0.001	5.3	4.7	6.0	0.06	25.9	<0.001	8.3	7.3	9.6	0.07	30.2	<0.001
*Paternal*	9.5	7.6	11.9	0.11	19.9	<0.001	9.4	7.6	11.6	0.11	21.1	&0.001	7.2	6.2	8.4	0.08	26.0	<0.001	11.2	9.6	13.1	0.08	30.3	<0.001
*Spouse*	1.0	1.0	1.0	0.00		<0.001	1.0	1.0	1.0	0.00		<0.001	1.0	1.0	1.0	0.00		<0.001	1.0	1.0	1.0	0.00		<0.001
*Other*	17.5	13.3	23.1	0.14	20.4	<0.001	16.5	12.6	21.6	0.14	20.4	<0.001	8.2	6.8	10.0	0.10	21.3	<0.001	16.2	13.3	19.6	0.10	28.3	<0.001
Any own child (binary)	1.0	1.0	1.0			<0.001	1.0	1.0	1.0			<0.001	1.0	1.0	1.0			<0.001	1.0	1.0	1.0			<0.001
Presence in household (all individual continuous variables)																								
*Under 1 year*	1.0	0.9	1.1	0.07	-0.1	0.934	1.0	0.9	1.1	0.07	-0.4	0.704	1.2	1.1	1.3	0.04	4.3	<0.001	1.2	1.1	1.3	0.05	3.8	<0.001
*1-4 years*	1.0	0.9	1.1	0.04	0.0	0.961	1.0	1.0	1.1	0.03	1.2	0.226	1.1	1.1	1.2	0.02	4.6	<0.001	1.0	1.0	1.1	0.03	1.4	0.164
*5-18 years*	0.9	0.9	1.0	0.02	-4.8	<0.001	0.9	0.8	0.9	0.02	-8.4	<0.001	0.9	0.9	0.9	0.01	-6.7	<0.001	0.9	0.9	0.9	0.01	-9.5	<0.001
*19-59 years*	0.9	0.8	0.9	0.03	-4.6	<0.001	0.8	0.7	0.8	0.03	-8.5	<0.001	0.9	0.9	0.9	0.02	-4.5	<0.001	0.9	0.9	1.0	0.02	-4.4	<0.001
*60 years and over*	0.8	0.7	0.9	0.06	-4.8	<0.001	0.7	0.7	0.8	0.05	-6.1	<0.001	0.9	0.8	0.9	0.04	-3.8	<0.001	0.8	0.8	0.9	0.04	-5.8	<0.001
Presence of family type within 250m (all individual binary variables)																								
*Maternal*	1.3	1.1	1.4	0.07	3.4	0.001	1.2	1.1	1.4	0.06	3.1	0.002	0.8	0.7	0.9	0.05	-4.7	<0.001	0.8	0.7	0.9	0.05	-4.3	<0.001
*Paternal*	1.0	0.9	1.2	0.07	0.5	0.620	1.1	1.0	1.2	0.06	1.5	0.126	0.9	0.8	0.9	0.05	-3.2	0.002	0.7	0.7	0.8	0.05	-6.6	<0.001
*Sister*	1.5	1.1	2.0	0.15	2.6	0.010	1.1	0.9	1.5	0.13	1.0	0.309	1.4	1.1	1.7	0.11	2.8	0.006	1.1	0.8	1.3	0.12	0.5	0.648
*Brother*	0.6	0.5	0.8	0.13	-3.9	<0.001	0.7	0.5	0.8	0.10	-4.2	<0.001	0.7	0.6	0.8	0.09	-4.2	<0.001	0.8	0.7	1.0	0.08	-2.5	0.013
*Nuclear*	1.3	1.1	1.5	0.08	3.1	0.002	1.3	1.2	1.5	0.07	4.6	<0.001	0.8	0.7	0.9	0.06	-4.3	<0.001	0.5	0.5	0.6	0.07	-9.1	<0.001
Maternal orphan (binary)	1.2	0.9	1.6	0.15	1.1	0.282	1.4	1.1	1.8	0.12	2.9	0.004	1.2	1.0	1.5	0.10	1.8	0.075	1.1	0.9	1.3	0.09	1.2	0.229
Paternal orphan (binary)	1.0	0.8	1.2	0.10	-0.2	0.870	1.1	1.0	1.3	0.08	1.6	0.109	0.9	0.8	1.0	0.07	-1.5	0.133	0.9	0.8	1.0	0.06	-2.1	0.039
Age (continuous)	1.1	1.1	1.1	0.01	7.9	<0.001	1.0	1.0	1.0	0.01	4.3	<0.001	1.2	1.2	1.2	0.01	25.9	<0.001	1.1	1.1	1.1	0.01	16.9	<0.001
Calendar year (categorical)																								
*2004-5*	0.8	0.6	0.9	0.10	-2.9	0.004	0.8	0.7	1.0	0.09	-2.0	0.042	0.6	0.5	0.6	0.07	-8.6	<0.001	0.5	0.5	0.6	0.07	-8.7	<0.001
*2006-7*	0.7	0.6	0.8	0.09	-3.8	<0.001	0.8	0.7	0.9	0.08	-3.0	0.003	0.7	0.6	0.8	0.06	-5.7	<0.001	0.7	0.6	0.8	0.06	-5.6	<0.001
*2008-9*	Reference						Reference						Reference						Reference					
*2010-11*	0.8	0.6	0.9	0.09	-3.2	0.001	0.9	0.7	1.0	0.08	-1.8	0.071	0.8	0.7	1.0	0.06	-2.8	0.005	0.8	0.7	0.9	0.06	-2.8	0.005
*2012-13*	0.8	0.7	0.9	0.09	-2.8	0.005	0.8	0.7	0.9	0.08	-3.3	0.001	0.8	0.7	0.9	0.06	-2.9	0.003	0.7	0.7	0.8	0.06	-4.8	<0.001
*2014-15*	0.7	0.6	0.9	0.09	-3.7	<0.001	0.8	0.7	0.9	0.08	-3.4	0.001	0.6	0.6	0.7	0.06	-7.2	<0.001	0.6	0.5	0.7	0.07	-8.4	<0.001
*2016-17*	0.7	0.6	0.9	0.09	-3.8	<0.001	0.7	0.6	0.8	0.08	-4.5	<0.001	0.5	0.4	0.6	0.07	-10.8	<0.001	0.5	0.4	0.5	0.07	-11.0	<0.001
Distance to tarmac road in km (continuous integer)	1.0	1.0	1.0	0.01	-2.7	0.008	1.0	1.0	1.0	0.01	-2.5	0.011	1.0	1.0	1.0	0.01	0.7	0.472	1.0	1.0	1.0	0.01	0.2	0.809
Population density within 250m (categorical)																								
*0.00150p*	1.2	1.0	1.3	0.08	2.0	0.045	1.1	1.0	1.3	0.07	1.8	0.064	1.1	1.0	1.2	0.05	2.2	0.025	1.1	1.0	1.3	0.05	2.6	0.009
*50-149p*	Reference						Reference						Reference						Reference					
*150-299p*	1.2	1.0	1.3	0.07	2.1	0.038	1.1	0.9	1.2	0.06	1.1	0.274	1.1	1.0	1.2	0.05	1.2	0.238	1.2	1.1	1.3	0.05	3.0	0.003
*>=300p*	1.0	0.9	1.2	0.08	0.1	0.948	0.9	0.8	1.1	0.08	-0.8	0.412	1.1	1.0	1.2	0.06	1.9	0.059	1.1	1.0	1.2	0.06	1.5	0.134
Father secondary education (categorical)																								
*None*	Reference						Reference						Reference						Reference					
*Secondary*	0.9	0.8	1.1	0.06	-0.8	0.398	1.1	1.0	1.2	0.06	1.5	0.143	1.0	1.0	1.1	0.04	1.0	0.339	0.9	0.8	1.0	0.05	-1.9	0.052
*Unknown*	0.9	0.6	1.4	0.21	-0.3	0.762	0.9	0.6	1.3	0.21	-0.6	0.559	3.5	2.9	4.3	0.10	12.5	<0.001	3.3	2.7	4.0	0.10	11.5	<0.001
Mother secondary education (categorical)																								
*None*	Reference						Reference						Reference						Reference					
*Secondary*	1.0	0.9	1.2	0.06	0.4	0.662	1.0	0.9	1.1	0.06	-0.1	0.940	1.0	0.9	1.1	0.05	-0.9	0.387	1.0	0.9	1.1	0.05	0.7	0.500
*Unknown*	0.4	0.2	0.8	0.29	-2.9	0.003	0.5	0.3	0.9	0.26	-2.4	0.018	1.0	0.8	1.2	0.12	-0.3	0.745	1.1	0.9	1.4	0.12	0.8	0.399
Household head employment score (categorical)																								
*Low*	0.9	0.8	1.2	0.12	-0.5	0.646	1.1	0.9	1.3	0.10	1.0	0.330	1.1	1.0	1.3	0.08	1.6	0.108	1.2	1.0	1.4	0.08	2.2	0.027
*Medium*	Reference						Reference						Reference						Reference					
*High*	0.9	0.8	1.1	0.08	-1.0	0.334	0.8	0.7	1.0	0.07	-2.6	0.009	1.0	0.9	1.1	0.05	-0.9	0.346	1.0	0.9	1.1	0.05	0.5	0.591
*Unknown*	2.9	2.1	4.2	0.18	6.0	<0.001	3.3	2.4	4.4	0.16	7.5	<0.001	1.2	0.9	1.6	0.15	1.3	0.202	1.3	1.0	1.7	0.14	1.9	0.055

**Table 4.** Results for short accompanied and long accompanied move outcome for children, from multi-level multinomial regression models. Models run separately for female children (aged <12 [19,650 individuals]) and male children (aged <16 [21,267 individuals]); outcome is move type with ‘no move’ as baseline; models allow for clustering by unique household id and unique participant id; short moves are under 4 kilometres; independent moves are without a parent of any age or an adult aged 18 or over

	Children (short accompanied moves)												Children (long accompanied moves)											
	Female (4841 moves)						Male (5421 moves)						Female (5062 moves)						Male (5740 moves)					
	OR	95% CI		SE	Z	p	OR	95% CI		SE	Z	p	OR	95% CI		SE	Z	p	OR	95% CI		SE	Z	p
Household composition																								
*Parents & siblings*	Reference						Reference						Reference						Reference					
*Sister's family*	0.7	0.5	0.9	0.14	-3.0	0.003	0.7	0.6	0.9	0.12	-2.8	0.004	0.8	0.6	1.0	0.14	-2.0	0.051	0.8	0.7	1.0	0.11	-1.9	0.059
*Brother's family*	0.8	0.5	1.6	0.32	-0.5	0.611	1.6	1.1	2.4	0.20	2.4	0.015	1.5	0.8	2.9	0.32	1.3	0.181	1.7	1.1	2.6	0.21	2.6	0.010
*Mother & siblings*	1.4	1.3	1.5	0.05	6.6	<0.001	1.6	1.4	1.7	0.05	9.9	<0.001	1.3	1.2	1.5	0.05	6.3	<0.001	1.3	1.2	1.5	0.05	6.6	<0.001
*Father & stepmother*	0.8	0.7	1.0	0.11	-1.8	0.076	0.8	0.7	1.0	0.08	-2.3	0.022	1.2	1.0	1.4	0.09	2.0	0.047	0.9	0.8	1.0	0.08	-1.6	0.116
*Maternal*	0.8	0.7	0.9	0.06	-4.1	<0.001	0.8	0.7	0.9	0.06	-4.5	<0.001	0.9	0.8	1.0	0.06	-2.0	0.044	0.8	0.8	0.9	0.06	-3.0	0.003
*Paternal*	0.4	0.3	0.6	0.16	-5.5	<0.001	0.5	0.4	0.6	0.13	-5.7	<0.001	0.5	0.4	0.6	0.15	-4.9	<0.001	0.4	0.3	0.5	0.14	-6.6	<0.001
*Other*	0.6	0.4	0.9	0.19	-2.7	0.007	0.5	0.4	0.8	0.20	-3.4	0.001	0.4	0.3	0.6	0.19	-4.5	<0.001	0.4	0.2	0.5	0.20	-5.1	<0.001
Presence in household (all individual continuous variables)																								
*Under 1 year*	1.1	1.0	1.2	0.04	2.4	0.014	1.2	1.1	1.2	0.04	4.1	<0.001	0.9	0.9	1.0	0.04	-2.1	0.037	0.9	0.8	1.0	0.04	-3.2	0.001
*1-4 years*	1.0	1.0	1.1	0.02	0.6	0.554	1.0	1.0	1.1	0.02	1.2	0.237	0.9	0.9	0.9	0.02	-4.4	<0.001	0.9	0.9	0.9	0.02	-5.2	<0.001
*5-18 years*	0.9	0.9	0.9	0.01	-10.4	<0.001	0.9	0.9	0.9	0.01	-9.2	<0.001	0.8	0.8	0.8	0.01	-19.2	<0.001	0.8	0.8	0.8	0.01	-18.7	<0.001
*19-59 years*	1.2	1.1	1.2	0.02	8.6	<0.001	1.1	1.1	1.2	0.02	7.6	<0.001	1.2	1.1	1.2	0.02	7.5	<0.001	1.1	1.1	1.2	0.02	7.3	<0.001
*60 years and over*	1.2	1.1	1.2	0.04	3.5	<0.001	1.1	1.0	1.1	0.04	1.3	0.206	0.9	0.9	1.0	0.04	-1.7	0.088	0.9	0.8	1.0	0.04	-3.0	0.003
Presence of family type within 250m (all individual binary variables)																								
*Maternal*	1.1	1.0	1.2	0.05	1.8	0.064	1.1	1.0	1.2	0.04	1.9	0.063	0.6	0.5	0.6	0.05	-11.5	<0.001	0.5	0.5	0.6	0.05	-12.6	<0.001
*Paternal*	0.6	0.5	0.6	0.04	-15.2	<0.001	0.6	0.5	0.6	0.04	-14.9	<0.001	0.5	0.5	0.5	0.04	-20.1	<0.001	0.5	0.5	0.5	0.03	-20.5	<0.001
*Sister*	0.8	0.6	1.0	0.13	-1.7	0.092	0.9	0.7	1.1	0.11	-1.3	0.181	0.8	0.6	1.0	0.14	-2.0	0.046	0.7	0.6	0.9	0.12	-2.5	0.013
*Brother*	0.8	0.7	0.9	0.09	-2.7	0.007	0.6	0.5	0.7	0.08	-6.6	<0.001	0.4	0.3	0.5	0.11	-8.1	<0.001	0.4	0.3	0.5	0.09	-10.7	<0.001
*Nuclear*	1.0	0.9	1.1	0.05	0.4	0.717	0.9	0.8	1.0	0.05	-2.8	0.005	0.8	0.8	0.9	0.05	-3.6	<0.001	0.9	0.8	0.9	0.05	-3.4	0.001
Maternal orphan (binary)	1.2	0.9	1.7	0.17	1.3	0.183	1.3	1.0	1.7	0.14	1.7	0.088	0.6	0.4	0.9	0.22	-2.5	0.013	0.9	0.7	1.2	0.15	-0.6	0.527
Paternal orphan (binary)	1.2	1.0	1.4	0.08	2.2	0.031	1.1	1.0	1.2	0.07	1.2	0.214	0.7	0.6	0.8	0.09	-4.6	<0.001	0.7	0.6	0.9	0.07	-4.2	<0.001
Age (continuous)	0.9	0.9	0.9	0.01	-19.5	<0.001	0.9	0.9	0.9	0.00	-22.5	<0.001	0.9	0.9	0.9	0.01	-14.6	<0.001	0.9	0.9	0.9	0.00	-17.5	<0.001
Calendar year (categorical)																								
*2004-5*	1.1	1.0	1.2	0.06	1.9	0.052	1.1	1.0	1.3	0.05	2.5	0.012	0.8	0.7	0.8	0.06	-5.1	<0.001	0.8	0.7	0.9	0.05	-4.9	<0.001
*2006-7*	1.0	0.9	1.1	0.06	0.5	0.650	1.0	0.9	1.1	0.05	-0.5	0.636	0.8	0.7	0.8	0.05	-5.2	<0.001	0.9	0.8	0.9	0.05	-3.1	0.002
*2008-9*	Reference						Reference						Reference						Reference					
*2010-11*	0.9	0.8	1.0	0.06	-1.3	0.211	0.8	0.8	0.9	0.05	-3.1	0.002	0.8	0.7	0.9	0.05	-4.2	<0.001	0.9	0.8	1.0	0.05	-2.4	0.015
*2012-13*	0.9	0.8	1.0	0.06	-1.8	0.070	0.9	0.8	1.0	0.05	-2.2	0.026	0.9	0.8	1.0	0.05	-2.3	0.022	0.9	0.9	1.0	0.05	-1.1	0.293
*2014-15*	1.0	0.9	1.1	0.06	-0.6	0.527	0.9	0.8	1.0	0.05	-2.5	0.014	0.8	0.8	0.9	0.05	-3.2	0.002	0.9	0.8	1.0	0.05	-2.3	0.024
*2016-17*	0.9	0.8	1.0	0.06	-1.4	0.172	0.9	0.8	1.0	0.05	-1.4	0.167	0.6	0.5	0.7	0.06	-9.5	<0.001	0.6	0.5	0.7	0.05	-9.6	<0.001
Distance to tarmac road in km (continuous integer)	1.0	1.0	1.0	0.01	-4.6	<0.001	1.0	1.0	1.0	0.01	-3.2	0.001	1.0	1.0	1.0	0.01	1.6	0.101	1.0	1.0	1.0	0.01	3.6	<0.001
Population density within 250m (categorical)																								
*<50p*	0.9	0.8	1.0	0.05	-1.6	0.101	0.8	0.7	0.9	0.05	-4.8	<0.001	1.0	0.9	1.1	0.04	0.0	0.988	0.9	0.8	1.0	0.04	-2.1	0.036
*50-149p*	Reference						Reference						Reference						Reference					
*150-299p*	1.2	1.1	1.4	0.04	5.0	<0.001	1.2	1.1	1.3	0.04	4.6	<0.001	1.1	1.0	1.2	0.04	2.8	0.005	1.0	1.0	1.1	0.04	1.2	0.221
*>=300p*	1.8	1.6	1.9	0.05	12.6	<0.001	1.7	1.6	1.9	0.04	12.8	<0.001	1.2	1.1	1.3	0.04	3.4	0.001	1.2	1.1	1.3	0.04	3.7	<0.001
Father secondary education (categorical)																								
*None*	Reference						Reference						Reference						Reference					
*Secondary*	1.1	1.0	1.2	0.04	2.2	0.027	1.0	1.0	1.1	0.04	1.2	0.248	1.0	0.9	1.1	0.04	-0.2	0.850	1.0	0.9	1.1	0.04	0.4	0.683
*Unknown*	0.7	0.5	1.0	0.17	-2.2	0.030	0.8	0.6	1.0	0.15	-1.8	0.079	4.0	3.3	4.8	0.10	13.7	<0.001	3.5	2.9	4.3	0.10	12.7	<0.001
Mother secondary education (categorical)																								
*None*	Reference						Reference						Reference						Reference					
*Secondary*	1.1	1.0	1.1	0.04	1.3	0.181	1.0	1.0	1.1	0.04	1.2	0.247	1.0	0.9	1.0	0.04	-1.1	0.286	1.0	0.9	1.0	0.04	-1.0	0.327
*Unknown*	1.6	1.2	2.3	0.18	2.8	0.006	1.7	1.3	2.3	0.16	3.4	0.001	0.8	0.7	1.1	0.11	-1.5	0.135	1.0	0.8	1.2	0.11	-0.2	0.826
Household head employment score (categorical)																								
*Low*	1.0	0.9	1.2	0.07	0.6	0.544	1.2	1.0	1.4	0.07	2.5	0.013	1.2	1.1	1.4	0.07	2.9	0.003	1.2	1.0	1.4	0.07	2.5	0.012
*Medium*	Reference						Reference						Reference						Reference					
*High*	1.3	1.2	1.4	0.04	6.5	<0.001	1.5	1.4	1.6	0.04	10.6	<0.001	1.7	1.5	1.8	0.04	13.4	<0.001	1.7	1.6	1.9	0.04	15.3	<0.001
*Unknown*	1.7	1.3	2.1	0.12	4.2	<0.001	1.7	1.4	2.1	0.11	4.7	<0.001	2.7	2.3	3.3	0.09	11.8	<0.001	3.2	2.8	3.8	0.08	15.0	<0.001

**Table 5.** Results for short independent and long independent outcome for adolescents, from multi-level multinomial regression models. Models run separately for female adolescents (aged 12–24 [13,748 individuals]) and male adolescents (aged 16–28 [10,208 individuals]); outcome is move type with ‘no move’ as baseline; models allow for clustering by unique household id and unique participant id; short moves are under 4 kilometres; independent moves are without a parent of any age or an adult aged 18 or over

	Adolescents (short independent moves)												Adolescents (long independent moves)											
	Female (4458 moves)						Male (2492 moves)						Female (6327 moves)						Male (3217 moves)					
	OR	95% CI		SE	Z	p	OR	95% CI		SE	Z	p	OR	95% CI		SE	Z	p	OR	95% CI		SE	Z	p
Household composition																								
*Parents & siblings*	Reference						Reference						Reference						Reference					
*Sister's family*	1.3	1.1	1.5	0.08	3.7	<0.001	1.0	0.9	1.2	0.09	0.4	0.682	1.6	1.4	1.7	0.06	7.7	<0.001	1.5	1.3	1.8	0.09	4.7	<0.001
*Brother's family*	1.9	1.6	2.3	0.10	6.4	<0.001	1.7	1.4	2.1	0.11	5.2	<0.001	1.7	1.4	2.0	0.09	5.9	<0.001	2.5	2.0	3.0	0.10	9.0	<0.001
*Mother & siblings*	1.4	1.2	1.6	0.06	5.4	<0.001	1.0	0.9	1.2	0.08	0.3	0.756	1.3	1.2	1.4	0.05	5.3	<0.001	1.5	1.3	1.8	0.08	5.5	<0.001
*Father & stepmother*	2.0	1.7	2.3	0.08	8.8	<0.001	1.6	1.4	1.9	0.09	5.7	<0.001	2.0	1.7	2.2	0.06	11.1	<0.001	2.0	1.7	2.4	0.08	8.3	<0.001
*Maternal*	1.9	1.6	2.1	0.07	8.3	<0.001	1.6	1.3	1.9	0.09	5.2	<0.001	2.1	1.9	2.3	0.06	12.9	<0.001	2.0	1.7	2.4	0.09	8.3	<0.001
*Paternal*	2.7	2.3	3.1	0.08	12.2	<0.001	1.7	1.4	2.1	0.10	5.5	<0.001	2.4	2.2	2.7	0.06	14.3	<0.001	2.4	2.0	2.9	0.09	9.4	<0.001
*Spouse*	0.3	0.3	0.4	0.07	-14.8	<0.001	0.2	0.2	0.3	0.12	-13.4	<0.001	0.3	0.3	0.4	0.06	-17.2	<0.001	0.7	0.6	0.9	0.11	-3.4	0.001
*Other*	1.7	1.4	1.9	0.08	6.0	<0.001	1.1	0.9	1.3	0.10	0.5	0.590	2.2	1.9	2.5	0.07	12.1	<0.001	3.2	2.7	3.8	0.09	13.5	<0.001
Any own child (binary)	0.8	0.7	0.9	0.08	-2.8	0.005	0.4	0.3	0.5	0.12	-8.1	<0.001	0.9	0.8	1.0	0.07	-2.2	0.027	1.0	0.8	1.2	0.11	-0.1	0.935
Presence in household (all individual continuous variables)																								
*Under 1 year*	1.0	1.0	1.1	0.04	1.1	0.260	1.1	1.0	1.3	0.06	1.9	0.059	0.9	0.8	0.9	0.04	-4.1	<0.001	1.1	1.0	1.2	0.05	1.1	0.279
*1-4 years*	1.0	1.0	1.1	0.02	1.6	0.103	1.0	0.9	1.1	0.03	-0.2	0.857	1.0	1.0	1.0	0.02	-0.3	0.786	0.9	0.9	1.0	0.03	-3.3	0.001
*5-18 years*	1.0	1.0	1.0	0.01	-0.1	0.894	1.1	1.0	1.1	0.01	4.9	<0.001	1.0	1.0	1.0	0.01	-2.8	0.006	1.0	1.0	1.0	0.01	-0.4	0.716
*19-59 years*	0.9	0.9	1.0	0.02	-3.1	0.002	0.8	0.8	0.9	0.02	-7.9	<0.001	1.0	1.0	1.0	0.01	-1.3	0.203	1.0	1.0	1.0	0.02	-0.4	0.696
*60 years and over*	0.9	0.8	1.0	0.04	-2.8	0.005	0.9	0.8	1.0	0.04	-2.1	0.032	0.9	0.9	1.0	0.03	-2.7	0.008	1.0	0.9	1.1	0.04	-0.1	0.898
Presence of family type within 250m (all individual binary variables)																								
*Maternal*	1.2	1.1	1.3	0.05	3.3	0.001	1.1	1.0	1.2	0.06	1.2	0.235	0.9	0.8	1.0	0.04	-2.1	0.032	0.9	0.8	1.0	0.05	-1.5	0.127
*Paternal*	1.0	0.9	1.1	0.04	0.8	0.445	1.0	0.9	1.1	0.05	-0.4	0.683	0.9	0.9	1.0	0.04	-2.4	0.015	0.8	0.7	0.8	0.05	-5.6	<0.001
*Sister*	1.1	1.0	1.3	0.06	2.2	0.028	0.9	0.8	1.1	0.06	-1.1	0.274	1.0	0.9	1.1	0.05	0.5	0.628	1.0	0.9	1.1	0.06	0.1	0.914
*Brother*	1.2	1.0	1.3	0.05	2.9	0.004	1.1	1.0	1.2	0.05	1.2	0.248	1.0	0.9	1.1	0.04	0.3	0.779	0.8	0.8	0.9	0.05	-3.8	<0.001
*Nuclear*	1.1	1.0	1.2	0.06	1.6	0.104	1.0	0.9	1.2	0.06	0.6	0.531	0.9	0.8	1.0	0.05	-2.2	0.030	0.7	0.6	0.7	0.07	-6.5	<0.001
Maternal orphan (binary)	1.2	1.0	1.4	0.08	2.5	0.012	1.1	0.9	1.3	0.08	0.8	0.440	1.1	1.0	1.2	0.06	1.6	0.103	1.1	0.9	1.2	0.07	0.7	0.487
Paternal orphan (binary)	1.0	0.9	1.1	0.05	-0.7	0.483	1.0	0.9	1.1	0.06	0.3	0.768	1.0	0.9	1.1	0.04	0.1	0.907	1.0	0.9	1.1	0.05	0.0	0.982
Age (continuous)	1.1	1.1	1.1	0.01	18.5	<0.001	1.1	1.1	1.1	0.01	17.3	<0.001	1.1	1.1	1.1	0.01	23.8	<0.001	1.1	1.1	1.1	0.01	14.4	<0.001
Calendar year (categorical)																								
*2004-5*	1.1	1.0	1.2	0.06	1.2	0.227	1.3	1.1	1.6	0.08	3.7	<0.001	0.7	0.6	0.7	0.05	-8.1	<0.001	0.5	0.4	0.5	0.07	-10.3	<0.001
*2006-7*	0.9	0.8	1.0	0.06	-2.0	0.044	1.1	1.0	1.3	0.08	1.6	0.099	0.8	0.7	0.9	0.05	-4.9	<0.001	0.6	0.6	0.7	0.07	-6.6	<0.001
*2008-9*	Reference						Reference						Reference						Reference					
*2010-11*	0.9	0.8	1.0	0.06	-2.2	0.031	0.8	0.7	1.0	0.09	-2.1	0.033	0.9	0.8	0.9	0.05	-3.4	0.001	0.9	0.8	1.0	0.06	-2.2	0.031
*2012-13*	0.8	0.7	0.9	0.06	-3.3	0.001	1.0	0.9	1.2	0.08	0.2	0.833	0.8	0.7	0.9	0.05	-5.1	<0.001	0.7	0.6	0.8	0.07	-4.6	<0.001
*2014-15*	0.7	0.6	0.8	0.06	-6.7	<0.001	1.1	0.9	1.2	0.08	0.6	0.518	0.7	0.6	0.7	0.05	-8.1	<0.001	0.8	0.7	0.9	0.06	-4.2	<0.001
*2016-17*	0.6	0.6	0.7	0.06	-7.9	<0.001	1.0	0.9	1.2	0.08	0.5	0.641	0.5	0.5	0.6	0.05	-12.8	<0.001	0.5	0.5	0.6	0.07	-9.2	<0.001
Distance to tarmac road in km (continuous integer)	1.0	1.0	1.0	0.01	-1.4	0.160	1.0	1.0	1.0	0.01	3.9	<0.001	1.0	1.0	1.0	0.01	3.8	<0.001	1.0	1.0	1.0	0.01	-1.0	0.301
Population density within 250m (categorical)																								
*<50p*	0.9	0.8	1.0	0.05	-2.7	0.006	1.0	0.9	1.1	0.06	0.3	0.795	1.1	1.1	1.2	0.04	3.3	0.001	1.1	1.0	1.2	0.05	1.4	0.164
*50-149p*	Reference						Reference						Reference						Reference					
*150-299p*	1.0	0.9	1.1	0.04	0.0	0.962	1.0	0.9	1.2	0.06	0.7	0.504	1.0	1.0	1.1	0.04	0.6	0.539	1.1	1.0	1.3	0.05	2.8	0.006
*>=300p*	0.9	0.9	1.0	0.05	-1.1	0.266	1.1	1.0	1.3	0.06	1.7	0.092	1.0	0.9	1.1	0.04	-0.5	0.643	1.1	1.0	1.3	0.06	2.2	0.026
Father secondary education (categorical)																								
*None*	Reference						Reference						Reference						Reference					
*Secondary*	1.0	0.9	1.1	0.04	0.0	0.975	0.9	0.8	1.0	0.05	-1.9	0.060	0.8	0.8	0.9	0.03	-5.6	<0.001	1.0	0.9	1.1	0.04	-0.2	0.870
*Unknown*	1.1	0.9	1.4	0.12	0.7	0.488	0.7	0.5	1.0	0.15	-2.2	0.030	2.2	1.9	2.5	0.08	10.3	<0.001	2.6	2.2	3.2	0.09	10.4	<0.001
Mother secondary education (categorical)																								
*None*	Reference						Reference						Reference						Reference					
*Secondary*	1.1	1.0	1.2	0.04	1.8	0.078	0.9	0.9	1.1	0.06	-1.0	0.314	0.9	0.8	0.9	0.04	-3.6	<0.001	0.9	0.8	1.0	0.05	-2.6	0.010
*Unknown*	0.7	0.5	0.9	0.14	-2.5	0.013	0.7	0.5	1.0	0.17	-1.9	0.062	1.7	1.5	2.0	0.08	6.6	<0.001	1.9	1.6	2.3	0.10	6.4	<0.001
Household head employment score (categorical)																								
*Low*	1.2	1.1	1.4	0.06	3.1	0.002	0.9	0.8	1.1	0.09	-1.0	0.325	1.1	1.0	1.2	0.06	1.9	0.064	1.2	1.0	1.3	0.07	2.0	0.045
*Medium*	Reference						Reference						Reference						Reference					
*High*	0.8	0.7	0.9	0.05	-4.1	<0.001	0.8	0.7	1.0	0.07	-2.5	0.011	0.9	0.8	1.0	0.04	-2.6	0.010	1.1	1.0	1.2	0.05	1.6	0.103
*Unknown*	1.7	1.3	2.2	0.14	3.7	<0.001	1.1	0.8	1.8	0.22	0.6	0.519	0.8	0.6	1.0	0.13	-2.1	0.033	1.1	0.8	1.5	0.15	0.7	0.476

**Table 6.** Results for short accompanied and long accompanied outcome for adolescents, from multi-level multinomial regression models. Models run separately for female adolescents (aged 12–24 [13,748 individuals]) and male adolescents (aged 16–28 [10,208 individuals]); outcome is move type with ‘no move’ as baseline; models allow for clustering by unique household id and unique participant id; short moves are under 4 kilometres; independent moves are without a parent of any age or an adult aged 18 or over

	Adolescents (short accompanied moves)												Adolescents (long accompanied moves)											
	Female (2039 moves)						Male (1378 moves)						Female (1892 moves)						Male (1321 moves)					
	OR	95% CI		SE	Z	p	OR	95% CI		SE	Z	p	OR	95% CI		SE	Z	p	OR	95% CI		SE	Z	p
Household composition																								
*Parents & siblings*	Reference						Reference						Reference						Reference					
*Sister's family*	0.8	0.6	1.1	0.14	-1.6	0.116	0.8	0.6	1.2	0.17	-1.1	0.285	1.0	0.8	1.3	0.12	0.3	0.781	0.8	0.6	1.1	0.14	-1.3	0.192
*Brother's family*	1.6	1.1	2.4	0.21	2.2	0.027	2.2	1.6	3.2	0.18	4.4	<0.001	1.2	0.7	1.8	0.22	0.7	0.512	1.2	0.8	1.7	0.18	0.9	0.376
*Mother & siblings*	2.1	1.7	2.5	0.09	7.7	<0.001	1.9	1.5	2.4	0.13	4.9	<0.001	1.0	0.8	1.2	0.10	-0.1	0.885	0.8	0.6	1.0	0.13	-1.9	0.059
*Father & stepmother*	1.5	1.1	1.9	0.13	3.0	0.002	1.8	1.4	2.4	0.15	4.1	<0.001	1.2	0.9	1.5	0.12	1.4	0.162	1.1	0.8	1.4	0.13	0.4	0.712
*Maternal*	1.0	0.8	1.2	0.12	-0.2	0.836	0.9	0.7	1.3	0.16	-0.5	0.595	0.6	0.5	0.8	0.13	-3.6	<0.001	0.6	0.4	0.8	0.16	-3.6	<0.001
*Paternal*	1.0	0.7	1.4	0.17	-0.1	0.903	1.7	1.2	2.4	0.18	2.9	0.003	0.6	0.5	0.9	0.16	-2.7	0.007	0.6	0.4	1.0	0.21	-2.1	0.035
*Spouse*	4.2	3.5	5.2	0.10	14.1	<0.001	5.6	4.2	7.5	0.15	11.9	<0.001	1.2	1.0	1.5	0.10	1.9	0.058	2.1	1.6	2.8	0.15	5.0	<0.001
*Other*	0.6	0.4	1.0	0.21	-2.1	0.040	0.9	0.5	1.4	0.24	-0.6	0.535	0.5	0.4	0.8	0.16	-3.7	<0.001	0.5	0.4	0.8	0.20	-3.1	0.002
Any own child (binary)	1.3	1.0	1.8	0.14	2.2	0.028	1.3	1.0	1.6	0.12	2.0	0.041	1.4	1.1	1.9	0.15	2.5	0.014	2.5	1.9	3.3	0.15	6.3	<0.001
Presence in household (all individual continuous variables)																								
*Under 1 year*	1.1	1.0	1.3	0.05	2.6	0.010	1.2	1.1	1.4	0.07	3.4	0.001	1.0	0.9	1.1	0.06	-0.6	0.539	1.0	0.9	1.1	0.07	-0.1	0.944
*1-4 years*	1.0	0.9	1.1	0.03	-0.1	0.955	1.0	0.9	1.1	0.04	-0.1	0.918	0.9	0.8	0.9	0.04	-3.5	0.001	0.9	0.8	1.0	0.05	-2.4	0.018
*5-18 years*	1.0	1.0	1.1	0.02	1.3	0.181	0.9	0.9	1.0	0.02	-2.5	0.013	0.9	0.9	0.9	0.02	-5.9	<0.001	0.9	0.9	0.9	0.02	-4.8	<0.001
*19-59 years*	1.2	1.1	1.3	0.02	7.3	<0.001	1.3	1.3	1.4	0.03	10.9	<0.001	1.2	1.1	1.2	0.03	5.7	<0.001	1.2	1.1	1.3	0.03	5.8	<0.001
*60 years and over*	1.1	1.0	1.2	0.06	1.6	0.111	1.2	1.1	1.4	0.07	2.8	0.006	0.9	0.8	1.0	0.07	-1.7	0.082	0.8	0.7	0.9	0.08	-3.1	0.002
Presence of family type within 250m (all individual binary variables)																								
*Maternal*	1.2	1.0	1.3	0.07	2.1	0.035	1.2	1.1	1.4	0.07	2.8	0.005	0.6	0.5	0.8	0.09	-4.9	<0.001	0.8	0.7	1.0	0.08	-2.5	0.013
*Paternal*	0.6	0.6	0.7	0.07	-6.1	<0.001	0.7	0.6	0.8	0.07	-5.0	<0.001	0.5	0.4	0.6	0.08	-9.6	<0.001	0.5	0.5	0.6	0.07	-9.0	<0.001
*Sister*	1.1	0.9	1.3	0.11	0.6	0.527	0.7	0.6	0.9	0.08	-3.7	<0.001	0.8	0.6	1.0	0.13	-2.0	0.046	0.8	0.7	1.0	0.09	-2.0	0.044
*Brother*	0.6	0.5	0.8	0.10	-4.5	<0.001	0.6	0.6	0.7	0.07	-6.6	<0.001	0.4	0.4	0.6	0.11	-7.2	<0.001	0.6	0.5	0.7	0.07	-7.0	<0.001
*Nuclear*	0.7	0.6	0.9	0.12	-2.6	0.010	0.7	0.6	0.9	0.12	-3.0	0.002	0.7	0.5	0.9	0.13	-2.8	0.005	0.5	0.4	0.7	0.13	-5.2	<0.001
Maternal orphan (binary)	1.2	0.9	1.5	0.12	1.3	0.207	0.9	0.7	1.2	0.12	-0.5	0.647	0.9	0.7	1.2	0.13	-0.6	0.581	1.0	0.8	1.3	0.12	-0.1	0.900
Paternal orphan (binary)	0.9	0.8	1.0	0.07	-1.3	0.188	1.0	0.8	1.1	0.08	-0.3	0.797	0.9	0.8	1.1	0.08	-0.7	0.467	0.9	0.8	1.1	0.08	-1.3	0.206
Age (continuous)	0.9	0.9	1.0	0.01	-5.6	<0.001	1.0	1.0	1.0	0.01	0.1	0.925	1.0	1.0	1.0	0.01	-0.1	0.903	1.0	1.0	1.0	0.01	-0.4	0.721
Calendar year (categorical)																								
*2004-5*	1.3	1.1	1.5	0.09	2.9	0.003	1.3	1.0	1.6	0.11	2.1	0.036	0.8	0.7	1.0	0.09	-2.1	0.033	0.6	0.5	0.8	0.11	-4.5	<0.001
*2006-7*	1.0	0.9	1.2	0.09	0.3	0.729	1.2	0.9	1.4	0.11	1.5	0.142	0.8	0.7	1.0	0.09	-1.9	0.052	0.8	0.6	1.0	0.10	-2.4	0.017
*2008-9*	Reference						Reference						Reference						Reference					
*2010-11*	1.0	0.8	1.2	0.09	0.0	0.975	1.0	0.8	1.3	0.11	0.4	0.667	0.9	0.7	1.0	0.09	-1.5	0.136	0.8	0.7	1.0	0.10	-1.7	0.095
*2012-13*	0.9	0.8	1.1	0.09	-0.7	0.469	1.1	0.9	1.3	0.11	0.6	0.564	0.9	0.7	1.1	0.09	-1.4	0.175	0.9	0.7	1.1	0.10	-1.3	0.206
*2014-15*	0.9	0.8	1.1	0.09	-1.1	0.264	1.0	0.8	1.2	0.11	-0.3	0.800	0.9	0.7	1.0	0.09	-1.7	0.085	0.8	0.6	1.0	0.10	-2.3	0.023
*2016-17*	1.0	0.8	1.2	0.09	-0.1	0.956	1.2	1.0	1.5	0.11	1.6	0.104	0.7	0.6	0.8	0.09	-4.5	<0.001	0.5	0.4	0.7	0.11	-5.6	<0.001
Distance to tarmac road in km (continuous integer)	1.0	1.0	1.0	0.01	-3.0	0.002	1.0	1.0	1.0	0.01	-0.7	0.464	1.0	1.0	1.0	0.01	1.4	0.153	1.0	1.0	1.0	0.01	-0.7	0.503
Population density within 250m (categorical)																								
*<50p*	0.8	0.7	1.0	0.08	-2.5	0.012	0.7	0.6	0.9	0.09	-3.3	0.001	0.8	0.7	1.0	0.07	-2.3	0.023	0.8	0.7	1.0	0.08	-2.0	0.049
*50-149p*	Reference						Reference						Reference						Reference					
*150-299p*	1.2	1.0	1.3	0.07	2.3	0.022	1.1	0.9	1.2	0.08	0.8	0.419	1.1	0.9	1.2	0.06	0.9	0.374	0.9	0.8	1.0	0.08	-1.5	0.128
*>=300p*	1.9	1.6	2.1	0.07	9.5	<0.001	1.6	1.3	1.8	0.08	5.7	<0.001	1.2	1.1	1.4	0.07	3.2	0.001	1.0	0.8	1.2	0.08	-0.2	0.844
Father secondary education (categorical)																								
*None*	Reference						Reference						Reference						Reference					
*Secondary*	1.0	0.9	1.1	0.06	-0.2	0.874	1.1	1.0	1.3	0.07	1.7	0.098	0.9	0.8	1.0	0.06	-2.7	0.006	0.9	0.8	1.1	0.07	-1.0	0.306
*Unknown*	1.2	0.8	1.6	0.16	0.9	0.359	0.8	0.6	1.2	0.19	-0.9	0.380	2.3	1.8	3.0	0.13	6.2	<0.001	2.3	1.7	3.0	0.15	5.5	<0.001
Mother secondary education (categorical)																								
*None*	Reference						Reference						Reference						Reference					
*Secondary*	0.9	0.8	1.0	0.06	-1.2	0.240	1.0	0.9	1.2	0.07	0.2	0.842	0.8	0.7	0.9	0.06	-4.4	<0.001	0.9	0.8	1.1	0.08	-0.9	0.373
*Unknown*	0.7	0.5	1.0	0.19	-1.9	0.062	0.9	0.6	1.4	0.22	-0.5	0.605	1.2	0.9	1.7	0.14	1.6	0.116	1.8	1.3	2.5	0.16	3.9	<0.001
Household head employment score (categorical)																								
*Low*	1.1	0.9	1.4	0.11	1.1	0.250	1.1	0.8	1.3	0.12	0.5	0.645	1.0	0.8	1.3	0.13	0.0	0.994	1.3	1.0	1.7	0.13	2.0	0.050
*Medium*	Reference						Reference						Reference						Reference					
*High*	1.9	1.7	2.1	0.06	10.7	<0.001	1.8	1.5	2.0	0.07	7.8	<0.001	2.4	2.2	2.7	0.06	15.6	<0.001	2.5	2.2	2.9	0.07	13.2	<0.001
*Unknown*	2.8	2.0	3.8	0.16	6.3	<0.001	2.1	1.3	3.2	0.23	3.3	0.001	5.7	4.5	7.2	0.12	14.6	<0.001	4.8	3.6	6.5	0.15	10.5	<0.001

### Household composition

**Figure 5.**
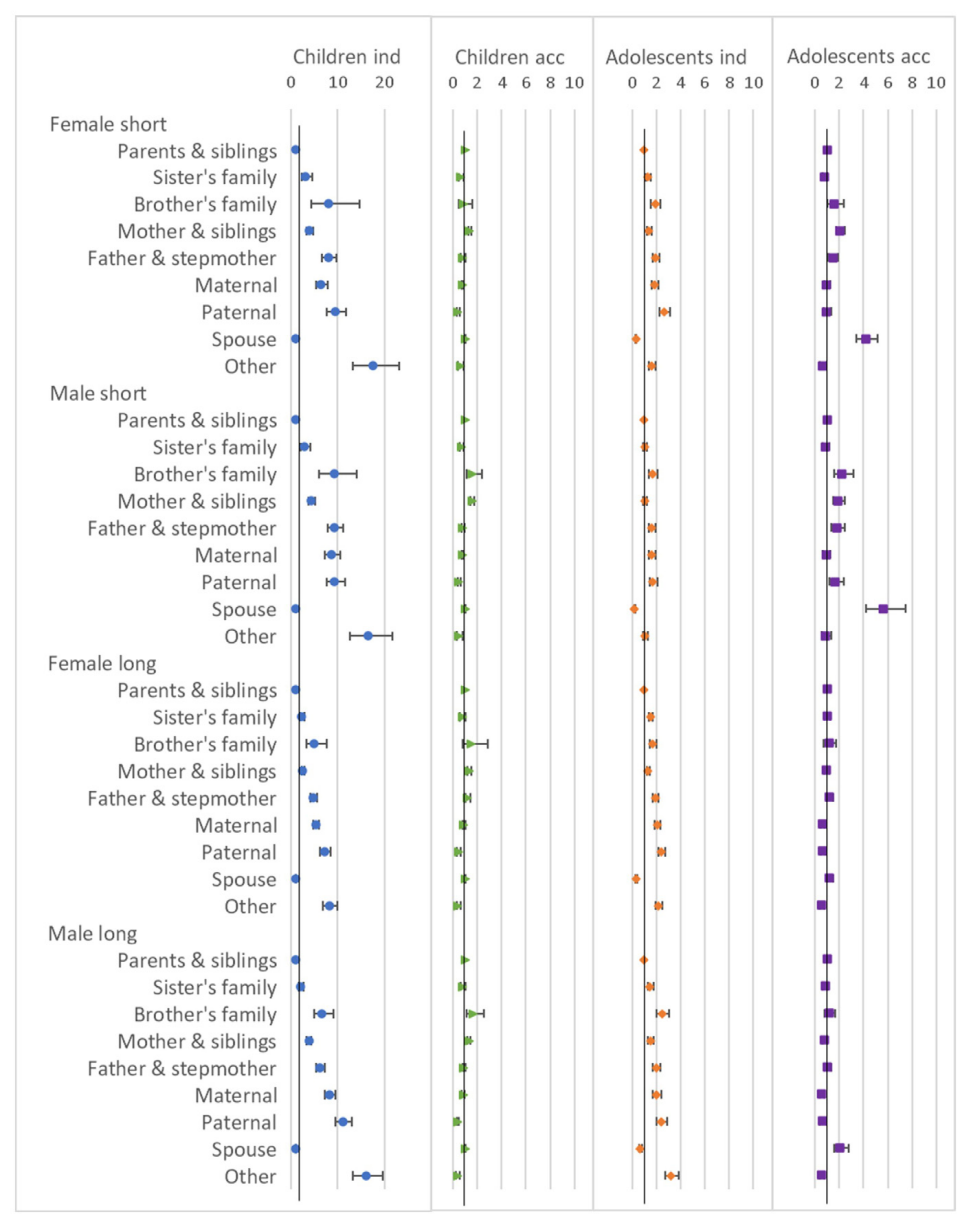
Odds ratios relating to sending household composition from multinomial multi-level regression models. The models run separately for female children (aged <12), male children (aged <16), female adolescents (aged 12–24) and male adolescents (aged 16–28); outcome is move type with ‘no move’ as baseline; models allow for clustering by unique household id and unique participant id; household composition is a single categorical variable with ‘parents & siblings’ as baseline; models control for presence of own child (adolescents only), household age composition, presence of family within 250 metres, orphanhood, age, year, distance to tarmac road, population density, parental education and household head employment score; short moves are under 4 kilometres; independent moves are without a parent of any age or an adult aged 18 or over; note the differing axes for ‘children ind’ compared to the others.

Results from the regression models for the sending household composition variable are shown in Figure 5. In all cases the baseline category was ‘ *parents and siblings’*. While there are associations between this variable and mobility for all age groups and types of move, the associations are strongest for children moving independently. For this group, the highest odds ratios tended to be for the ‘ *other’* category (meaning children living in this category were more likely to move out than those living with *‘parents and siblings’*), followed by ‘ *paternal’*, ‘ *brother’s family’*, ‘ *maternal’* and ‘ *father and stepmother’*. For child accompanied moves, the odds ratios were closer to 1 than for the independent moves, suggesting the composition of the sending household was less strongly associated with mobility for accompanied than independent moves, and the associations were more varied: ‘ *sister’s family’*, ‘ *paternal’* and ‘ *other’* were associated with slightly lower odds of moving and ‘ *mother and siblings’* with slightly higher odds. The odds ratios for adolescents moving independently followed a similar pattern to those of the children, albeit closer to 1. The ‘ *spouse’* household type, which was not present for children, is the only category associated with lower odds of move, suggesting adolescents were less likely to leave *‘spouse’* households independently than *‘parents and siblings’* household. The odds ratios for adolescent accompanied moves tended to be closer to 1, and somewhat similar to the patterns seen for children; for this move type the *’spouse’* category was mostly strongly associated with higher odds of moving, for all except female long moves.

### Household age composition

**Figure 6.**
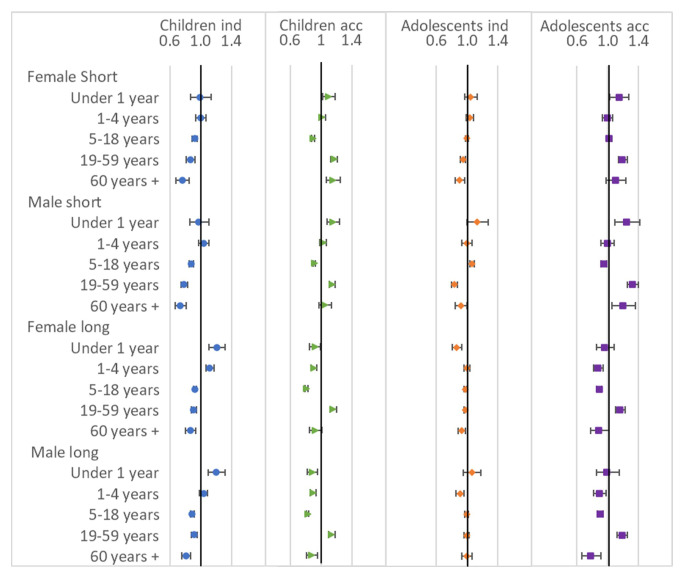
Odds ratios relating to sending household age composition from multinomial multi-level regression models. The models run separately for female children (aged <12), male children (aged <16), female adolescents (aged 12–24) and male adolescents (aged 16–28); outcome is move type with ‘no move’ as baseline; models allow for clustering by unique household id and unique participant id; household age composition are all individual variables of total number in each age group [excluding index] in the household; models control for household composition, presence of own child (adolescents only), presence of family within 250 metres, orphanhood, age, year, distance to tarmac road, population density, parental education and household head employment score; short moves are under 4 kilometres; independent moves are without a parent of any age or an adult aged 18 or over.

Results from the regression models for the household age composition variables are shown in Figure 6. For children moving independently, presence of increasing number of children aged under 5 in the household either has no association or was associated with higher chance of moving; while increasing number of people aged five and over was associated with lower chance of move, with the odds ratios for the number of people aged 60 years+ the furthest from 1 (suggesting the lowest chance of moving). The effects were different for child accompanied moves, with increasing number of children aged 18 and under having no association or associated with a lower chance of move; increasing number of 19–59-year-olds was associated with higher odds of move; and increasing number of adults aged 60 or over was associated with higher odds for short move but lower odds for long moves. For adolescents moving independently there was not such a clear pattern, but increasing numbers of other household members tended to be associated with lower odds of move: for long moves presence of children aged under one year (but not older children) was associated with lower chance of move for females; however for males this association was seen with one-four year-olds (but not younger children). For accompanied adolescents, increasing numbers of 19–59-year-olds was consistently associated with higher chance of move, while, for long moves, all other age groups (children and those aged 60+) were associated with lower chance of move.

### Individual relative variables

**Figure 7.**
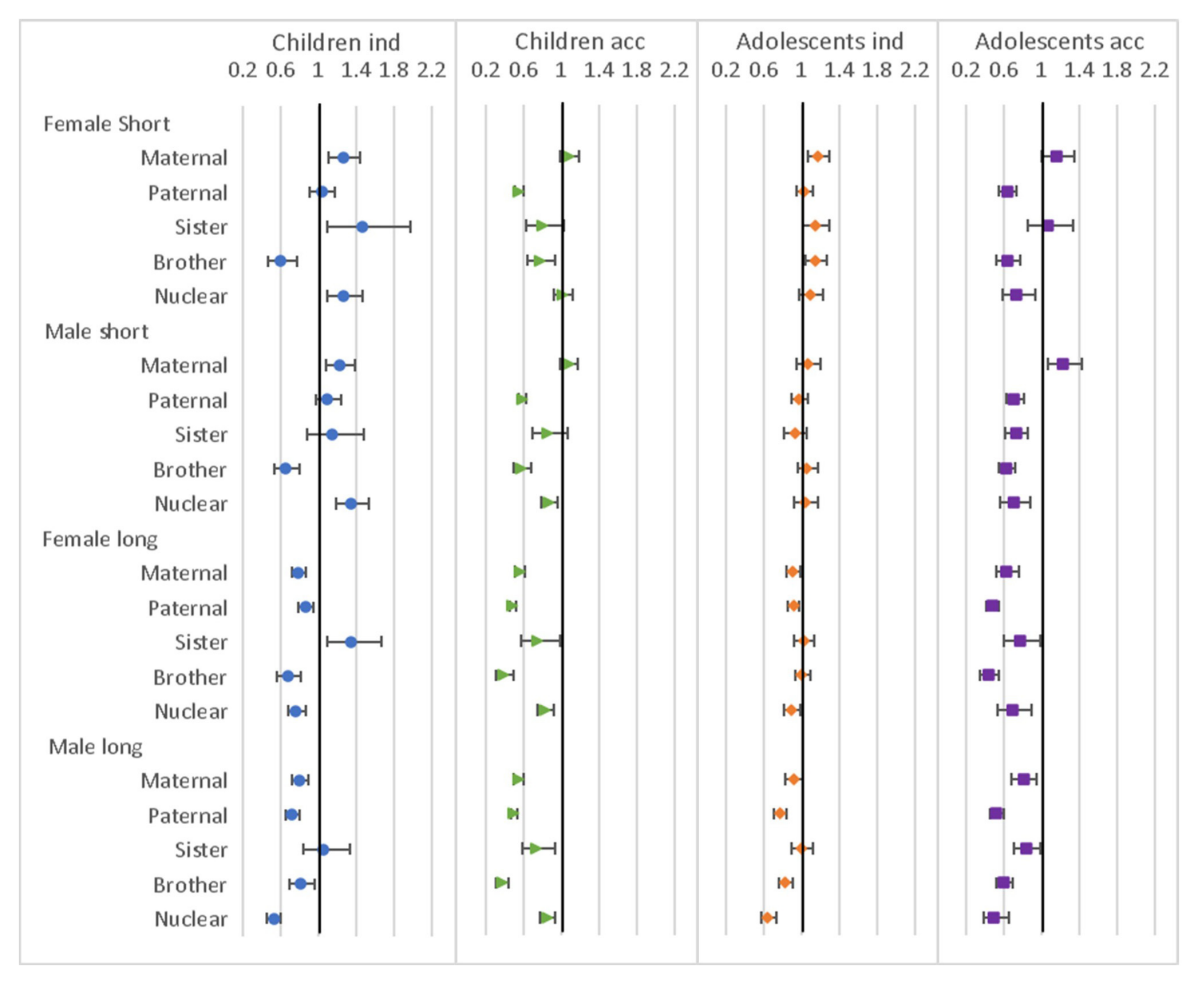
Odds ratios relating to family living nearby from multinomial multi-level regression models. The models run separately for female children (aged <12), male children (aged <16), female adolescents (aged 12–24) and male adolescents (aged 16–28); outcome is move type with ‘no move’ as baseline; models allow for clustering by unique household id and unique participant id; family nearby are all individual binary variables; models control for household composition, presence of own child (adolescents only), household age composition, orphanhood, age, year, distance to tarmac road, population density, parental education and household head employment score; short moves are under 4 kilometres; independent moves are without a parent of any age or an adult aged 18 or over.

Results from the regression models for the presence of family within 250 metres (but not in the household) variables are shown in Figure 7. For children moving independently, presence of maternal and nuclear family (and sister’s family for female children only) were associated with higher chance of move, while brother’s family was associated with a lower chance. For long moves the pattern was different with all family types associated with lower chance of move, except sister, which was associated with higher chance for females. For accompanied moves, there was either an association with lower chance of move for all family types, or, in few cases, no association. For the adolescents moving independently, the odds ratios tended to be closer to 1, particularly for short moves, but the pattern for male long moves was similar to that of the children. For accompanied moves most family types were associated with lower chance of move, except maternal for short moves.

### Other factors

**Figure 8.**
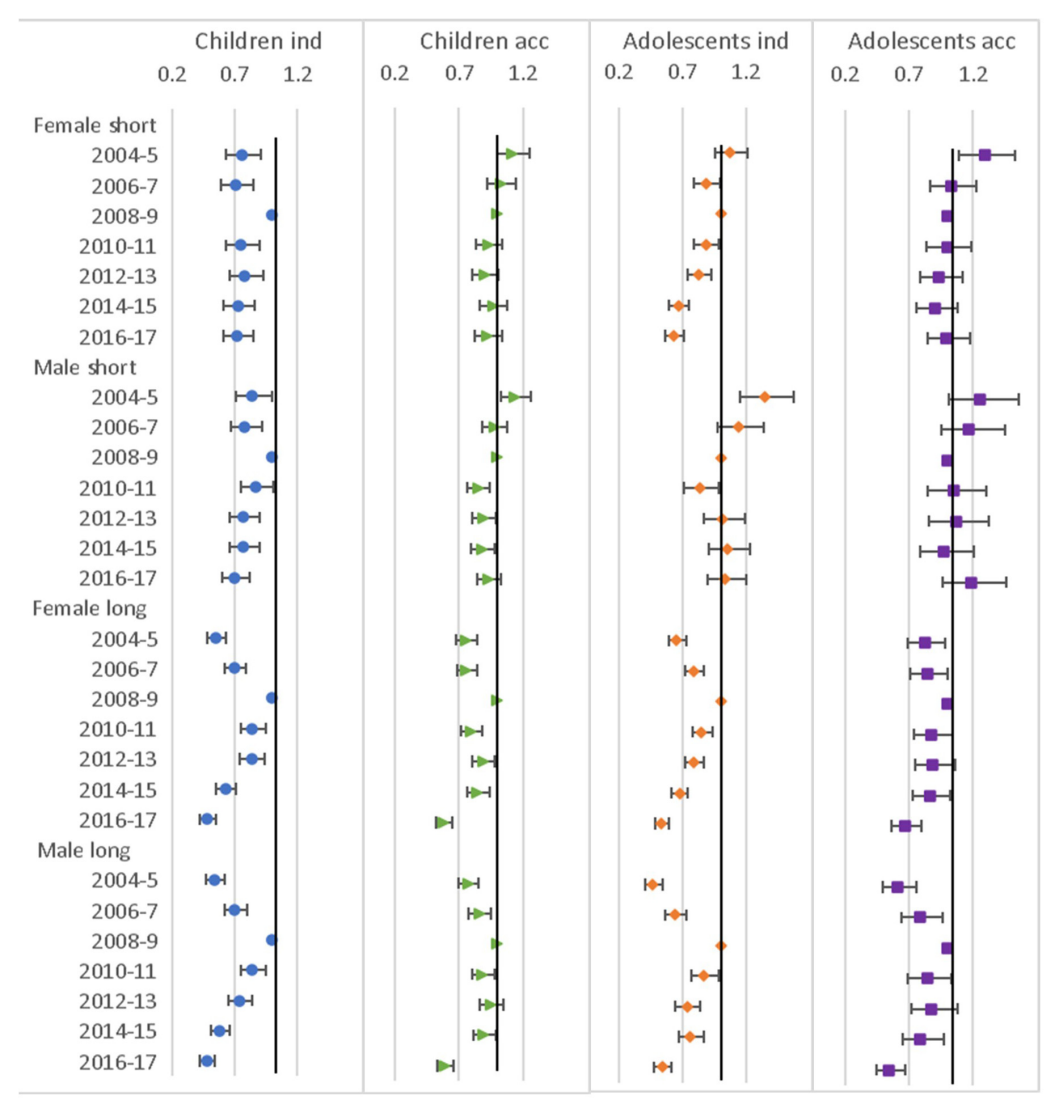
Odds ratios relating to calendar year from multinomial multi-level regression models. The models run separately for female children (aged <12), male children (aged <16), female adolescents (aged 12–24) and male adolescents (aged 16–28); outcome is move type with ‘no move’ as baseline; models allow for clustering by unique household id and unique participant id; calendar year is in 2-year categories with 2008–9 as baseline; models control for household composition, presence of own child (adolescents only), household age composition, presence of family within 250 metres, orphanhood, age, distance to tarmac road, population density, parental education and household head employment score; short moves are under 4 kilometres; independent moves are without a parent of any age or an adult aged 18 or over.

**Figure 9.**
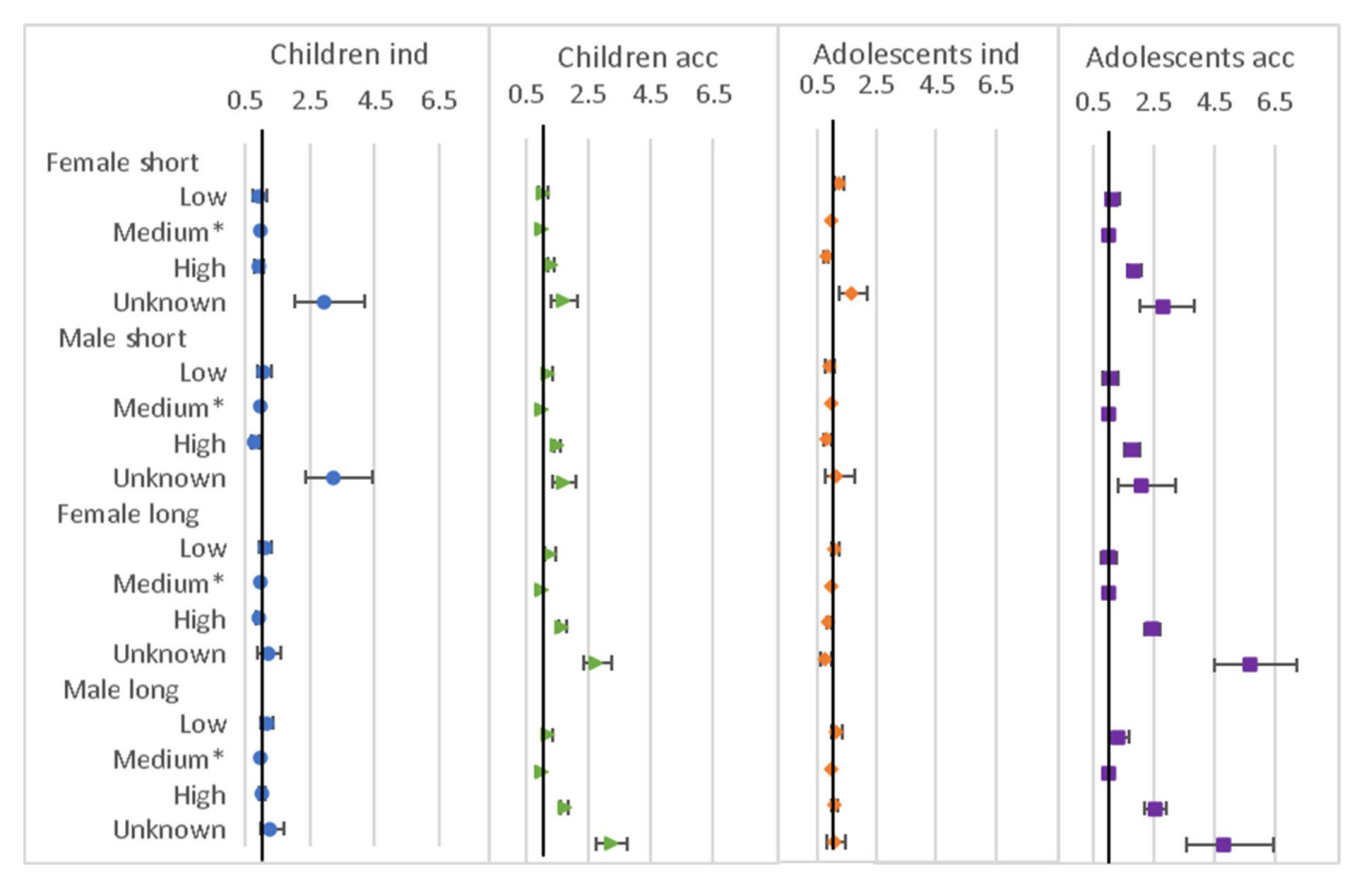
Odds ratios relating to household head employment score from multinomial multi-level regression models. The models run separately for female children (aged <12), male children (aged <16), female adolescents (aged 12–24) and male adolescents (aged 16–28); outcome is move type with ‘no move’ as baseline; models allow for clustering by unique household id and unique participant id; household head employment score is in 3 categories with ‘medium’ as baseline; models control for household composition, presence of own child (adolescents only), household age composition, presence of family within 250 metres, orphanhood, age, year, distance to tarmac road, population density and parental education; short moves are under 4 kilometres; independent moves are without a parent of any age or an adult aged 18 or over.

The effect of calendar year is clearest for the long moves for both children and adolescents and short and long moves, where there was less chance of moving both before and after the baseline period of 2008–9, and the odds of moving getting smaller with later calendar time. For short moves, there was a similar pattern (though closer to 1) for children moving independently but for the other groups it was less clear ( Figure 8). The employment level of the household head of the sending household had little effect on independent moves, though for short independent child moves those with unknown status were more likely to move. For accompanied moves those in the high and unknown category had higher odds of moving compared to the medium category, this was particularly the case for long moves and adolescents ( Figure 9).

## Discussion

### Summary of findings

This analysis on mobility in rural Malawi shows two key periods of mobility in very young childhood and adolescence/young adulthood which relate to leaving home and marriage as part of the transition to adulthood, and young couples moving to establish themselves in the community while their children are young. The tradition of patrilocality can be seen in these data, with young women moving to join their spouses, however the culture of children ‘belonging’ to the paternal family is less clear with children seeming to be treated differently according to their sex. Moving seems to be strongly linked to local presence of family, in that the presence of family living nearby but not in the same household tends to be associated with lower chances of moving. Mobility is also related to socio-economic status and appears to become less common over time.

### Moving is common for young families

The high level of movement in young families can be seen in the high rates of accompanied move for infants and young children, which sharply, and then more gradually, decrease until about the age of 12. High move rates in young children have been found elsewhere ( Ford & Hosegood, 2005; Grieger *et al.*, 2013). There is also an increase in accompanied moves in young adulthood. Evidence from the regression modelling shows that adolescents living in a ‘ *Spouse’* household type are more likely to move accompanied ( *i.e.* with their spouse) and that presence of very young children was associated with short accompanied moves but not long ones, implying that parents with young families are likely to move around locally but not to migrate long distances. Also, not living with a parent ( *i.e.*, with *paternal* household) tended to be associated with lower chance of move than living in a *parents & siblings* household. The descriptive analysis shows that children moving accompanied often do not experience a change in household type implying it is a relocation of the household, rather than it breaking up, although there is also evidence of accompanied moves due to marital dissolution ( *i.e.* children commonly move from ‘ *Parents and siblings*’ households to ‘ *Mother and siblings*’). A limitation of this dataset is that it is known that men who marry and start a family while still living with their parents are likely to declare themselves a distinct household so, if/when they did move away from the grandparents the move from *paternal* households to *parents and siblings* would not be observed in this data. 

### Adolescents move as part of transition to adulthood

Both short and long independent move risks rise during adolescence: this rise happens earlier than for accompanied moves and is also earlier for females than males. This is expected given that it is known that women tend to transition to adulthood earlier in this population ( McLean *et al.*, 2021b), a pattern which has been found elsewhere ( Beegle & Poulin, 2013; Ford & Hosegood, 2005; Grieger *et al.*, 2013). The patrilocal tradition can be observed as female adolescents are much more likely to leave the spousal home to return to the home of another relative (or leave the area), presumably following separation or divorce. Following a breakdown in a marriage, it appears that the men are likely to stay put. Both males and females had a higher chance of independent move when living in household types other than ‘ *parents and siblings’* (excluding *spouse*-type household): a study from another area of Malawi also found that adolescents living with parents were less likely to move than those with a more distant relationship to the household head ( Beegle & Poulin, 2013).

Presence of infants aged under one year was associated with lower chance of long independent move for female adolescents, which might be expected if they were ‘held back’ from their own marriage to care for other young children: for males this effect was also found for presence of one-four-year-old children. It may be that adolescents of both sexes delay long-distance moves to support young children in their households.

### Sex differences and cultural aspects

The most obvious difference between male and female children in these data is the higher rates of long independent moves for girls compared to boys from the age of four. We have found previously that there are sex differences in which relatives children and adolescents live with, if not with both parents: boys are more likely to live in ‘male’ relative household ( *i.e.* with brother, father without mother, or paternal relatives) while girls may be more likely to live with a sister ( McLean *et al.*, 2021a). This was confirmed in this analysis of mobility where some household moves were common for each sex, *i.e.,* girls were more likely to move to a *maternal* household: as the mother’s family is more likely to be further away (due to the patrilocal traditions) this may explain some of the difference in risk of long independent moves, however unfortunately we do not know what type of household most long-distance movers go to. These girls may also be fostered out to households better able to care for them: this is relatively common in sub-Saharan Africa and has been found to be slightly more likely for girls than boys in other settings ( Hedges *et al.*, 2019), or maybe being sent to help out in other households either doing house work or caring duties, which also tends to be more likely for girls ( Robson, 2000). Presence of small children was associated with higher chance of long independent move for female children, but not for males, indicated that the female children might be being moved out to make room for younger ones: a study in Malawi found that children were more likely to be fostered out if the mother had just had a baby, but that this depended on the mother’s marital status ( Grant & Yeatman, 2014).

Children of both sexes have a much higher rate of independent move in certain household types, generally when the mother was not present, which has also been observed elsewhere ( Madhavan *et al.*, 2012). It was also found that girls moving accompanied were more likely than boys to be moving with their mother: this may be because when a marriage breaks down and the woman moves out, she may be more likely to take the female children rather than the male.

In general, presence of relatives nearby is associated with lower chance of moving, indicating that family is a strong force in decision-making in this area. Although in this area children traditionally ‘belong’ to their paternal family, these data clearly show that the maternal family plays an important role. There were more moves to *maternal* households than *paternal* ones, and presence of maternal family nearby but not in the household is associated with higher chance of short move but lower chance of long move indicating that presence of maternal family ‘keeps’ families living in the same area. Living in a ‘ *Mother & siblings’* household is associated with higher chance of short and long accompanied move, and the descriptive analysis shows that the short moves at least tend to be relocations ( *i.e.*, remaining ‘ *Mother & siblings’*) or could be the mother and children moving to live with her family ( *i.e.*, *maternal* household), so it may be likely that a proportion of the long moves will be due to the mother returning to her home village. In contrast living in ‘ *Father and step-mother’* household (which will also include single father) was only associated with higher chance of short accompanied move and most of these are relocations ( *i.e.*, remaining ‘ *Father & step-mother* type), although it is likely that a man moving back to his parent’s property would maintain that he is a separate household so we wouldn’t capture this move in this data, it seems likely that if fathers do move following widowhood or divorce it is to elsewhere in the area.

### Other factors relating to mobility

Although family is clearly important in decision making about mobility, our analyses also found independent effects of other factors. Moving rates appear to be decreasing since 2008. There could be many reasons for this. It is known that transition to adulthood is becoming later for both sexes with adolescents more likely to stay in school longer before leaving home and marrying ( McLean *et al.*, 2021b), which would affect rates of mobility of young people. This area was also heavily affected by HIV/AIDS but increasing access to ART since the mid-2000s has reduced mortality rates ( Price *et al.*, 2017) which will have kept more households intact so children and adolescents have not had to move. Equally, fertility rates have dropped in the area ( McLean *et al.*, 2017) so if children and adolescents are moving to free up space in the household this will be required less. There does not appear to be a trend of young people increasingly moving to cities, as might be expected given increasing urbanisation and access to internet/media which has been found in other settings ( Hertrich & Lesclingand, 2012). Further analysis once more data become available are needed to assess whether the decrease is a lasting trend.

Household head socio-economic position seems to have little effect on independent move, but those in the most socio-economically advantaged group are most likely to move accompanied. This may be because those in the medium socio-economic group will tend to be farmers whose work is linked to their land, so it would be harder to move, while those in the most advantaged group may move due to work. A study in another area of Malawi also found that adolescents in the wealthiest households were most likely to move ( Beegle & Poulin, 2013), and in urban South Africa moving was more likely for children living at the top or bottom of the socio-economic scale ( Ginsburg *et al.*, 2009).

### Strengths and limitations

This analysis benefitted from a very detailed dataset capturing short and long distance moves with the ability to differentiate between independent and accompanied moves. However, the census is carried out annually, so short-term migrations occurring between the census rounds may be missed. Moves may be wrongly classified as independent or accompanied if move dates of other household members are wrongly captured (though collapsing the data into quarters reduces the reliance on the exact dates being the same) or, for example, if a person is joining a household member who moved shortly before. In addition, external moves may be more likely to be erroneously classed as accompanied as they only need to have reported moving from/to the same town/city rather than to the exact same house. GPS data for households within the area allow for calculations of distances moved and the ability to class people as living near certain relatives. Outside of the HDSS the move distances are estimated, however this should not have resulted in too many misclassifications into short/long as almost all external moves would be classed as long.

Short-term temporary moves or holidays would not be captured in our data: a study in Zambia found that a lot of children spent long periods of their school holidays staying with relatives, often helping out with chores they speculate that for some of these children these ‘holidays’ may be the result of a balancing act to allow the children to support the extended family but to keep them in school, whereas otherwise they might be fostered out more permanently ( Hunleth *et al.*, 2015).

Family links allow for very detailed analysis of the effects of presence of certain types of family, both in the household and nearby. However, family links are not available for all participants; this is most likely for people who have recently moved into the area, *i.e.* women who have moved into the area for marriage will be less likely to appear in the regression model due to being less likely to have at least one parent ID. Even if the individuals have parental IDs available, all their relatives may not, meaning that they will appear to have fewer relatives living nearby. Participants who have moved in will be less likely to have parental IDs, and if a previous move is related to a later move this may cause bias in the estimations. In our analysis, we assume that family members nearby will be in contact and will give and receive support in some manner which may result in them influencing migration decisions, however this may not be the case, and people without family may receive the same support from non-related people which might attenuate any effects of the presence of family.

Further factors beyond what was examined in the model might have an effect but were not available for all participants over the whole time period: missing data from surveys is likely to be associated with the outcome as more mobile households/individuals are more likely to be missed (as was observed in the household socio-economic status variable used in the model). In-depth interpretation of all the factors included in the model was also beyond the scope of this paper. This analysis used only data from single time snapshots just before the move or just after. As most moving decisions would be made over a longer period, examining exposures over a longer period before the move may provide greater understanding, however due to the nature of HDSS data this would not be possible.

## Conclusion

Using detailed longitudinal data from a rural HDSS in northern Malawi, we have shown that mobility is very common among young people. While some of these moves are clearly household relocations, children not uncommonly move independently of their parents, with 20% of moves involving unaccompanied children. Overall, we find considerable complexity in movement patterns, though some trends emerge. For example, sex differences in mobility are noticeable, with girls being more likely to move than boys from the age of four; and, in terms of age patterns, we replicate previous findings of high mobility for very young children and adolescents. But we also find complexity in the households which young people move to and from, and that the maternal family is clearly important in this traditionally patrilocal community. We further find that moves become less common over calendar time, and that we don’t observe an increasing trend for young people to move to more urban areas, as might be expected from a context of rapid urbanisation, however it has previously be shown that increasing urbanisation can be mostly explained by natural population increases in urban areas and reclassification of rural areas, rather than rural-urban migration ( Farrell, 2017).

## Data Availability

Due to the nature of the dataset (containing exact GPS coordinates of individuals households and potentially unique patterns of local relatives), it would not be possible to anonymise it in such a way that would sufficiently protect the participants’ privacy and allow for useful analyses. MEIRU are, however, keen to share data and collaborate with bona fide researchers and students at universities and research institutes. Interested parties should contact the first author [EM] through info@meiru.mw in the first instance, quoting the paper title. After a discussion of data needed, a signed data transfer agreement would be required. Metadata on the MEIRU dataset which formed the basis of these analyses can be found, along with information on other studies, on MEIRU’s data catalogue ( http://kpsmw.lshtm.ac.uk/nada/index.php/catalog/13), **Zenodo: Local and long-distance migration among young people in rural Malawi: importance of age, sex and family (author-written code).** Author-written code in Stata, R and MLwiN for article published on Wellcome Open Research. https://zenodo.org/record/7797357#.ZCvY6_bMJD8. **This project contains the following files:** Prog1_create_migration_datasets.do A Stata do-file containing code to manipulate the main dataset into the analytical dataset Prog2_explo_desc_analysis.do A Stata do-file containing code for the exploratory and descriptive analyses reported in this paper Prog3_prepare_forR_Sankey.do A Stata do-file containing code to manipulate the analytical dataset into the format required to generate the Sankey diagrams in R Prog4_prepare_forMLwinN_Model.do A Stata do-file containing code to manipulate the analytical dataset into the format required to run the multi-level modelling using MLwiN Prog5_sankey_plots.R An R script containing code to generate the Sankey diagrams reported in this paper Prog6_MLwiN_models.txt An MLwiN macro containing code to run the multi-level models reported in this paper Data are available under the terms of the Creative Commons Attribution 4.0 International
