# Supplementary material for: Local and long-distance migration among young people in rural Malawi: importance of age, sex and family
Source: Wellcome Open Res. 2024 Mar 18;8:211. Originally published 2023 May 11. [Version 2] doi: 10.12688/wellcomeopenres.19309.2 (PMC11109702; doi:10.12688/wellcomeopenres.19309.2)
Supplement: Peer review history

# Reviewer report: Reviewer response for version 2

**DOI:** 10.21956/wellcomeopenres.23515.r79846

**Published:** 2024-06-13

**Authors:** Carren Ginsburg

**Affiliations:** 1 University of Witswatersrand, Johannesburg, South Africa

This article presents descriptive analysis of migration of young people between 2004–2017 using longitudinal data from the Karonga Health and Demographic Surveillance System (HDSS).

 The strength of this article is the maximal and comprehensive use of available data to classify movement and integrate information on household composition (also determining whether moves were accompanied or independent). The study adds valuable evidence to the body of research on child and young adult mobility in transitional societies where movement may be independent, prompted by circumstances relating to a child/young adult or accompanied due to circumstances relating to a caregiver or connected adult.

 The study has a descriptive objective and confirms trends that have been observed in other studies, while offering some new insights on age and sex differences in movement and the importance of family in this setting. But the article is somewhat lacking in articulating how the findings may be used to guide further research or what the implications of these findings may be for policy or planning. Several studies on child migration and household composition have examined how these dynamics affect child health (e.g. mortality) or educational attainment/ progression. In relation to transition to adulthood, where movement is often driven by the search for employment, access to further education or marriage, migration may have diverse impacts on health and well-being.  It would be useful to have a broader set of conclusions or implications reported for the current study, and directions for future research that touch on some of these potential next steps.

 A few other suggestions and comments are offered below:

 The justification for the choice of age categories for males and females is based on earlier research and is supported in the age-sex distributions of move types in Figure 2 (this can be made more explicit in the Methods section, or description of Figure 2). A limitation of using different age groups for males and females is that they are not directly comparable although the authors’ state that these capture the different timing of transition to adulthood between males and females. It would be helpful to understand this better in relation to average ages of school completion and marriage for males and females in this population.

 The definition of accompanied moves – moves with a parent or an adult over the age of 18 – appears more applicable to child movement and less so to movement of young adults where the upper age limit is 24 for females and 28 for males. The rationale for the analysis of accompanied versus independent movement among young adults could be elaborated, in particular given more missing data resulting from a lack of parent identifiers in these ages.

 There are a few places in the article where the term “shorter” may be misunderstood as shorter duration rather than shorter distance. Since migration has a time and spatial dimension, it would be useful to make this distinction clear.

 Finally, in the case of short distance moves, it is not entirely clear whether these are necessarily movements that are internal to the HDSS boundary. A sentence of clarification on this could be added to the Methods section.

Is the work clearly and accurately presented and does it cite the current literature?

Yes

If applicable, is the statistical analysis and its interpretation appropriate?

Yes

Are all the source data underlying the results available to ensure full reproducibility?

Partly

Is the study design appropriate and is the work technically sound?

Yes

Are the conclusions drawn adequately supported by the results?

Partly

Are sufficient details of methods and analysis provided to allow replication by others?

Yes

Reviewer Expertise:

Internal migration, health, Health and Demographic Surveillance Systems, sub-Saharan Africa

I confirm that I have read this submission and believe that I have an appropriate level of expertise to confirm that it is of an acceptable scientific standard.

# Reviewer report: Reviewer response for version 2

**DOI:** 10.21956/wellcomeopenres.23515.r79845

**Published:** 2024-06-12

**Authors:** Chantel F. Pheiffer

**Affiliations:** 1 Population Studies and Training Center, Brown University, Providence, Rhode Island, USA; 2 Urban Public Health, University of Massachusetts Boston (Ringgold ID: 14708), Boston, Massachusetts, USA

This manuscript uses rich HDSS data from Malawi to explore patterns and predictors of migration among children and young adults. This paper is thorough, well-written, and presents a welcome contribution to our understanding of internal migration dynamics in low- and middle-income countries. 

 Literature Review: Is there a way to reconcile/elaborate on the two statements that a)  girls and young women are more likely to move; b) elders tend to be more reluctant to let girls move. Is it that girls move despite this reluctance, or that this reluctance only surrounds independent moves and that girls are more likely to be “sent” to move? It would be helpful to add a sentence or two to clarify this

 Measurement: Distance Measure: It is not clearly explained why the authors use where children move/attend school to determine the cutoff value to classify long vs. short moves in their analysis. A 4km move seems like a very short move, leaving the a huge range of “long” moves. Have the authors considered alternative cutoffs, or a sub-classification of mid vs. long-distance moves? 

 Data: Missingness- This analysis reports a lot of dropped observations - this is mentioned in the limitations section, but a more extensive discussion about whether/how the authors attempted to deal with missing data (is imputation possible for intermittent missingness?) and to discuss the implications or potential biases this introduces into the analysis would be useful.

 Methods: multiple testing- Have the authors considered adjusting the alpha levels in order to take into account multiple testing and multiple comparisons? 

 Substantive: marriage migration- Marriage migration is discussed in the literature review, but is not much discussed in the results and conclusion. It would be useful to know what average age of first marriage for women and girls in this population is, and whether/to what extent higher risk of moving among young women is attributable to marriage. 

 Minor:

 - It would be helpful to have the number of observations displayed for each group in Figure 2 rather than having to go back to Figure 1 or the text to get this information. 

 - Typo: “Generation some of the exposure variables…” (in “Dataset” section)

Is the work clearly and accurately presented and does it cite the current literature?

Yes

If applicable, is the statistical analysis and its interpretation appropriate?

Yes

Are all the source data underlying the results available to ensure full reproducibility?

No

Is the study design appropriate and is the work technically sound?

Yes

Are the conclusions drawn adequately supported by the results?

Yes

Are sufficient details of methods and analysis provided to allow replication by others?

Yes

Reviewer Expertise:

social demography; social determinants of health; internal migration; gender; South Africa

I confirm that I have read this submission and believe that I have an appropriate level of expertise to confirm that it is of an acceptable scientific standard.

# Reviewer report: Reviewer response for version 2

**DOI:** 10.21956/wellcomeopenres.23515.r79639

**Published:** 2024-05-09

**Authors:** Nan Zhang

**Affiliations:** 1 The University of Manchester, Manchester, England, UK

This manuscript concerns an important topic on migration patterns in rural Malawi in relation to age, sex and family dynamics. The paper is nicely written with active engagement with wider literature. The handling of data and methods are appropriate. The results are informative and reported nicely. I would like to see more in the discussion chapter about the significance of the findings by placing this piece of study based in Malawi in a wider or global context. It can be important to make better sense of your findings by comparing your findings with other countries that are undergoing the similar socioeconomic and demographic transitions in Africa and Asia, and what are the potential policy or theoretical implications of your findings?

 Abstract—Results and conclusions: It would be good to touch on explicitly the potential theoretical and policy implications or significance of the findings.

Is the work clearly and accurately presented and does it cite the current literature?

Yes

If applicable, is the statistical analysis and its interpretation appropriate?

Yes

Are all the source data underlying the results available to ensure full reproducibility?

Yes

Is the study design appropriate and is the work technically sound?

Yes

Are the conclusions drawn adequately supported by the results?

Partly

Are sufficient details of methods and analysis provided to allow replication by others?

Yes

Reviewer Expertise:

Ageing, migration, care, health

I confirm that I have read this submission and believe that I have an appropriate level of expertise to confirm that it is of an acceptable scientific standard.

# Reviewer report: Reviewer response for version 2

**DOI:** 10.21956/wellcomeopenres.23515.r77016

**Published:** 2024-03-28

**Authors:** Philippe Bocquier

**Affiliations:** 1 Centre de Recherches en Démographie, Universite catholique de Louvain, Louvain-la-Neuve, Walloon Region, Belgium

The revisions of the paper are good and carefully accounted for suggestions, in particular age is now better controlled for, figures are made comparable (coefficients on the same scale), residual categories were discarded, some figures were removed from the main paper giving it a clearer focus. 

 In the web version (but strangely not in the downloadable PDF version) the Figure 6 is a repeat of Figure 5. Figure 6 should have age and sex composition in rows.

Is the work clearly and accurately presented and does it cite the current literature?

Yes

If applicable, is the statistical analysis and its interpretation appropriate?

Partly

Are all the source data underlying the results available to ensure full reproducibility?

Partly

Is the study design appropriate and is the work technically sound?

Partly

Are the conclusions drawn adequately supported by the results?

Yes

Are sufficient details of methods and analysis provided to allow replication by others?

Yes

Reviewer Expertise:

Event history analysis, migration, mortality

I confirm that I have read this submission and believe that I have an appropriate level of expertise to confirm that it is of an acceptable scientific standard.

# Reviewer report: Reviewer response for version 1

**DOI:** 10.21956/wellcomeopenres.21392.r57166

**Published:** 2023-05-22

**Authors:** Philippe Bocquier

**Affiliations:** 1 Centre de Recherches en Démographie, Universite catholique de Louvain, Louvain-la-Neuve, Walloon Region, Belgium

This report provides an extensive examination of the factors influencing migration among children and adolescents up to the age of 24, with a detailed breakdown of household categories. The analysis is systematically carried out by sex, large age groups, and duration of migration. However, the abundance of tables and graphs and associated intepretations can be overwhelming at times, and the paper lacks a specific research question. It could have placed more emphasis on exploring either gender differences, or variations based on the duration of migration, or distinctions between independent and accompanied migration.

 My main concerns revolve around the methodology employed in the study:

- Despite the longitudinal nature of the data, the authors chose to employ a logit model with age controlled as a continuous variable. Considering the non-linear age effect, particularly after the age of 12, a Cox model or any other proportional hazards model for censored data would have been more suitable. The non-linearity of age effect is contradictory with the use of a parametric model such as the logit model.
- All figures presenting odds ratios should be given on a log scale, and it would be preferable to maintain consistent minimum and maximum values across the four categories crossing children/adolescents with accompanied/independent. This would allow for better visual interpretation of the data and facilitate comparisons between the categories.
- It would have been more effective to exclude from the figures the categories such as " *other*" for household composition or " *unknown*" for household head employment score. These residual categories lack specific interpretations and often exhibit high migration rates. Migration likely generates unclear status of household composition or employment.

To summarize, I believe the paper possesses relevance, but its lack of a clear focus is a notable shortcoming and makes it quite descriptive. Several methodological choices could have been made to sharpen the focus.

Is the work clearly and accurately presented and does it cite the current literature?

Yes

If applicable, is the statistical analysis and its interpretation appropriate?

Partly

Are all the source data underlying the results available to ensure full reproducibility?

Partly

Is the study design appropriate and is the work technically sound?

Partly

Are the conclusions drawn adequately supported by the results?

Yes

Are sufficient details of methods and analysis provided to allow replication by others?

Yes

Reviewer Expertise:

Event history analysis, migration, mortality

I confirm that I have read this submission and believe that I have an appropriate level of expertise to confirm that it is of an acceptable scientific standard, however I have significant reservations, as outlined above.
